# NMDAR mediated translation at the synapse is regulated by MOV10 and FMRP

**DOI:** 10.1186/s13041-019-0473-0

**Published:** 2019-07-10

**Authors:** Preeti Madhav Kute, Sarayu Ramakrishna, Nagammal Neelagandan, Sumantra Chattarji, Ravi. S. Muddashetty

**Affiliations:** 1 Centre for Brain Development and Repair (CBDR), Institute for Stem Cell Science and Regenerative Medicine (inStem), Bangalore, 560065 India; 2 School of Chemical and Biotechnology, Shanmugha Arts, Science and Technology & Research Academy (SASTRA) University, Thanjavur, 613401 India; 3 National Centre for Biological Sciences (NCBS), Bangalore, 560065 India; 4 The University of Trans-Disciplinary Health Sciences and Technology, Bangalore, 560064 India; 5 Centre for Discovery Brain Sciences, Deanery of Biomedical Sciences, University of Edinburgh, Edinburgh, EH89XD UK

**Keywords:** FMRP/MOV10/NMDAR mediated translation

## Abstract

Protein synthesis is crucial for maintaining synaptic plasticity and synaptic signalling. Here we have attempted to understand the role of RNA binding proteins, Fragile X Mental Retardation Protein (FMRP) and Moloney Leukemia Virus 10 (MOV10) protein in N-Methyl-D-Aspartate Receptor (NMDAR) mediated translation regulation. We show that FMRP is required for translation downstream of NMDAR stimulation and MOV10 is the key specificity factor in this process. In rat cortical synaptoneurosomes, MOV10 in association with FMRP and Argonaute 2 (AGO2) forms the inhibitory complex on a subset of NMDAR responsive mRNAs. On NMDAR stimulation, MOV10 dissociates from AGO2 and promotes the translation of its target mRNAs. FMRP is required to form MOV10-AGO2 inhibitory complex and to promote translation of MOV10 associated mRNAs. Phosphorylation of FMRP appears to be the potential switch for NMDAR mediated translation and in the absence of FMRP, the distinct translation response to NMDAR stimulation is lost. Thus, FMRP and MOV10 have an important regulatory role in NMDAR mediated translation at the synapse.

**Electronic supplementary material:**

The online version of this article (10.1186/s13041-019-0473-0) contains supplementary material, which is available to authorized users.

## Introduction

In mature neurons, protein synthesis in the dendrites and spines outweigh that of the cell body due to their sheer volume [1]. Protein synthesis at dendrites and spines is regulated by the activation of many different neurotransmitter receptors such as glutamate, dopamine, and serotonin [2–4] also termed as activity mediated protein synthesis. Thus, it is important to decipher the specificity of translational response to a given neurotransmitter receptor stimulation. This task has gained significance since the dysregulation of protein synthesis is thought to be a common cause for multiple neurodevelopmental disorders [5]. Glutamate is the major excitatory neurotransmitter in the mammalian brain and NMDAR and the group I metabotropic Glutamate Receptor (mGluR) are two of its primary receptors that mediate synaptic plasticity. Both NMDAR and mGluR regulate protein synthesis, group I mGluR leading to global translation activation and NMDAR to translation inhibition shown through metabolic labelling of proteins [6–9]. At transcriptome level, both group I mGluR and NMDAR stimulation leads to translation activation of specific subset of mRNAs. Group I mGluR stimulation leads to translation of mRNAs such as Fragile X mental retardation 1 (Fmr1), postsynaptic density 95 (Psd-95), activity regulated cytoskeleton associated protein (Arc) [10–12] and NMDAR stimulation leading to translation of β-actin, Glutamate receptor ionic epsilon 1 (Grin2a), Fmr1, Calcium/calmodulin dependent kinase II alpha (camk2a) and Arc mRNAs [9, 13–17]. Group I mGluR mediated translation activation is well studied, however, the mechanistic insight of NMDAR mediated translation is poorly explored [18, 19]. In the current study, we tried to elucidate NMDAR mediated control over the translation machinery by determining the factors involved in it.

MicroRNAs and microRNA induced silencing complex (miRISC) are thought to play an important role in regulating activity mediated protein synthesis. MicroRNA-AGO2 mediated translation inhibition can be reversed by dissociation of miRISC from the mRNA and promoting its translation [11, 20, 21]. This reversibility of miRISC is of particular interest in the context of synaptic plasticity as it can inhibit translation until an appropriate stimulus relieves the inhibition. Under these conditions, microRNAs provide the sequence specificity while several RNA binding proteins (RBP) which are not part of miRISC core complex will act as a molecular switch through their dynamic interaction with AGO2. FMRP is one such RBP which has a significant role in synaptic protein synthesis. Previously, it was shown that FMRP along with AGO2 regulate translation in response to the group I mGluR stimulation at the synapse [11]. While FMRP is also reported to regulate translation through multiple mechanisms [22, 23], its role in reversibility of miRISC mediated inhibition is likely to be of relevance for synaptic translation. The loss of FMRP and the subsequent synaptic dysfunction is the hallmark of Fragile X Syndrome (FXS) [24]. Interestingly, FMRP is reported to interact with a large number of mRNAs [25] and thus potentially regulates translation beyond mGluR signalling. Another RBP known to regulate translation downstream of synaptic signalling is MOV10 and is also known to interact with both FMRP and AGO2 [19, 26]. Since both NMDAR and group I mGluR mediated plasticity involve protein synthesis, it is also essential to study the role of FMRP and MOV10 in NMDAR mediated protein synthesis at the synapse.

In the current study, we show that the dynamic interaction between AGO2-MOV10-FMRP determines the translation response to NMDAR stimulation. This study highlights the involvement of FMRP and its phosphorylation status in NMDAR mediated signalling and provides a molecular mechanism to explain the specificity of translation on NMDAR stimulation.

## Results

### MOV10 dissociates from AGO2 and moves to polysomes on NMDAR stimulation

**Fig. 1:**
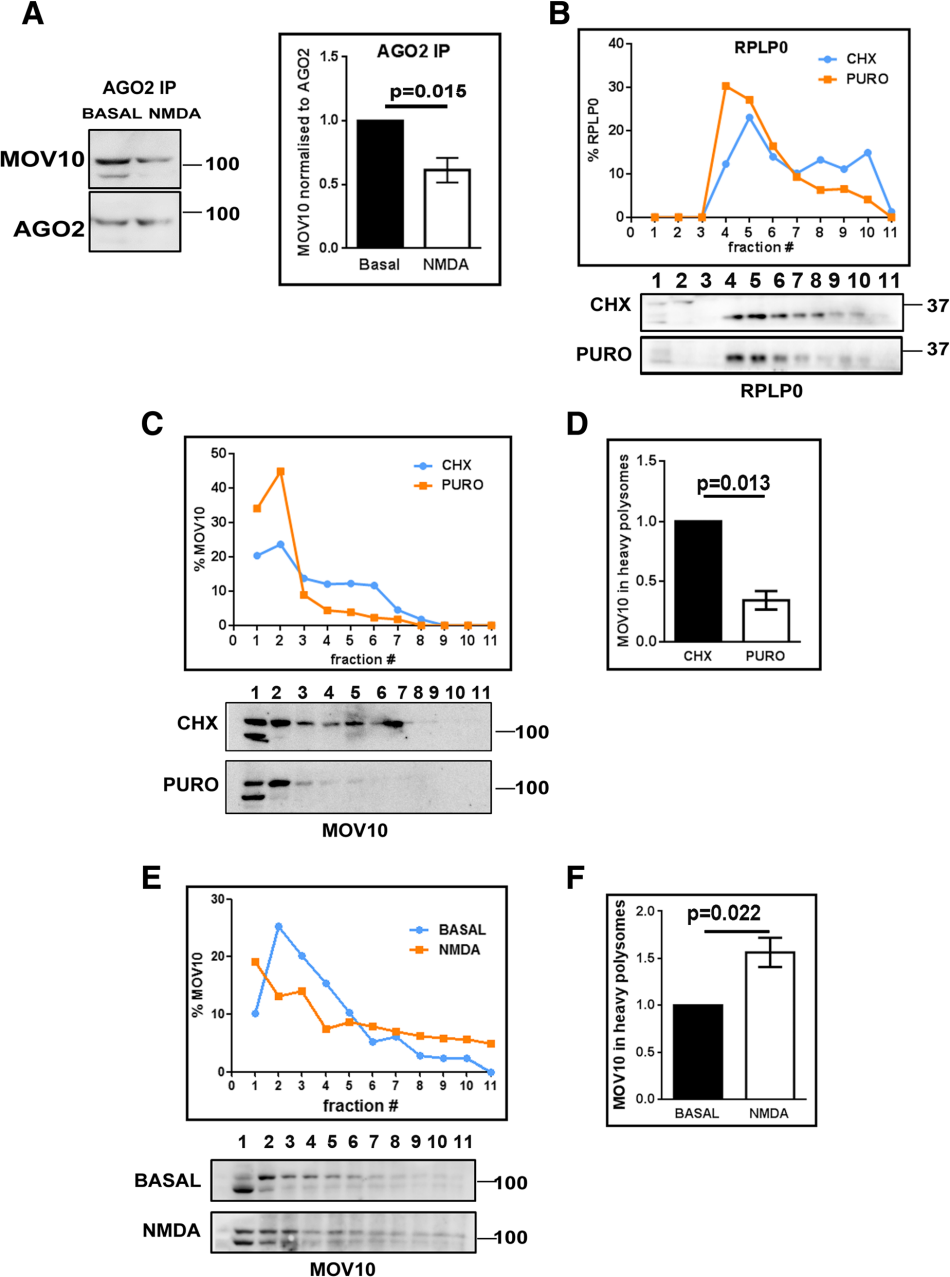
Effect of NMDAR stimulation on MOV10 interaction with AGO2 and distribution in polysomes. **a** Immunoblots for MOV10 and AGO2 after AGO2-immunoprecipitation on NMDAR stimulation. Quantitative analysis of MOV10 association with AGO2 following AGO2 immunoprecipitation on NMDAR stimulation in rat cortical synaptoneurosomes (*n* = 5, paired Student’s t-test, ±SEM). Values are normalized to basal levels. **b** Distribution of RPLP0 on linear sucrose gradient from rat cortical synaptoneurosomes after cycloheximide or puromycin treatment based on immunoblots shown below. **c** Distribution of MOV10 on linear sucrose gradient from rat cortical synaptoneurosomes after cycloheximide or puromycin treatment based on immunoblots shown below (representative of three experiments, also see Additional file 1: Figure S2A). **d** Quantification of MOV10 in heavy polysomes (fraction 7–11) for cycloheximide or puromycin treatment (*n* = 3, paired Student’s t-test, ±SEM). Values are normalised to cycloheximide levels. **e** Distribution of MOV10 on linear sucrose gradient from rat cortical synaptoneurosomes after NMDAR stimulation based on immunoblots shown below (representative of five experiments, also see Additional file 1: Figure S2B). **f** Quantification of MOV10 in heavy polysomes (fraction 7–11) for NMDAR stimulation as compared to basal condition (*n* = 5, paired Student’s t-test, ±SEM). Values are normalised to basal levels

In order to understand the mechanism of NMDAR mediated translation, we chose to investigate the role of MOV10 because of its implication in previous studies [19, 27]. MOV10 is an RNA helicase and is also shown to regulate the translation of its target mRNAs [19, 27, 28] Though MOV10 is proposed to play a role in NMDAR mediated translation in these studies, the molecular mechanism was not clear. In order to characterize its regulatory role, we looked at the association of MOV10 with miRISC protein AGO2 and with polysomes. We used post-natal day 30 (P30) rat cortical synaptoneurosomes for this study. Synaptoneurosomes were characterised by electron microscopy for the presence of postsynaptic density (PSD) and synaptic vesicles (SV) and for the enrichment of PSD-95 protein (Additional file 1**:** Figures S1A and S1B). The synaptoneurosome preparation used here is based on rapid filtration method. This method results in a relatively crude prep of synaptoneurosomes which are intact and are responsive to neurotransmitter stimulation [6, 11]. This preparation is suitable for our work since the focus is to study stimulation mediated changes in translation. We show that MOV10 co-precipitates with AGO2 from cortical synaptoneurosomes preparation (Additional file 1: Figure S1C). Further, we did AGO2 immunoprecipitation (IP) from synaptoneurosome lysate after NMDAR stimulation and quantified MOV10 association with AGO2 through western blot analysis (densitometric MOV10 values were normalized to that of AGO2). On NMDAR stimulation, there was a significant decrease in the association of MOV10 with AGO2 compared to basal (Fig. 1a) while there was no change in the levels of MOV10 (input) on NMDAR stimulation in synaptoneurosomes (Additional file 1: Figure S1E). These results hold true when we reversed the IP. For this, we did MOV10 IP, and looked for AGO2 in the pellet on NMDAR stimulation (Additional file 1: Figure S1D). We observed a decrease in the association of MOV10 and AGO2 on NMDAR stimulation, confirming that MOV10 dissociates from the inhibitory complex (AGO2) on NMDAR stimulation.

To understand the role of MOV10 on synaptic translation, we looked at the association of MOV10 protein with polysomes in synaptoneurosomes. In synaptoneurosomes, on puromycin (PURO) treatment, actively translating polysomes shift to lighter fractions compared to cycloheximide (CHX) treatment, as shown by ribosomal protein lateral stalk subunit P0 (RPLP0) (Fig. 1b). MOV10 was present in polysomal fractions but puromycin treatment led to significant reduction of MOV10 from heavy polysomes and a shift to lighter fractions (Fig. 1c, d and Additional file 1: Figure S2A) indicating that MOV10 is associated with actively translating polysomes. MOV10 distribution in polysomes was further validated using a sucrose step gradient method [29] in Neuro 2a cells **(**Additional file 1: Figures S2C-S2E**)**. Thus, we found that MOV10 associates with AGO2 as well as with puromycin sensitive polysomes. Interestingly, in synaptoneurosomes, the percentage of MOV10 in translating polysomes (puromycin-sensitive) was significantly increased on NMDAR stimulation compared to basal condition (Fig. 1e, f**,** Additional file 1**:** Figures S2B and S2F). These results show that on NMDAR stimulation MOV10 dissociates from inhibitory protein AGO2 and moves into translating polysomes.

### FMRP is required for the translation response downstream of NMDAR stimulation

**Fig. 2:**
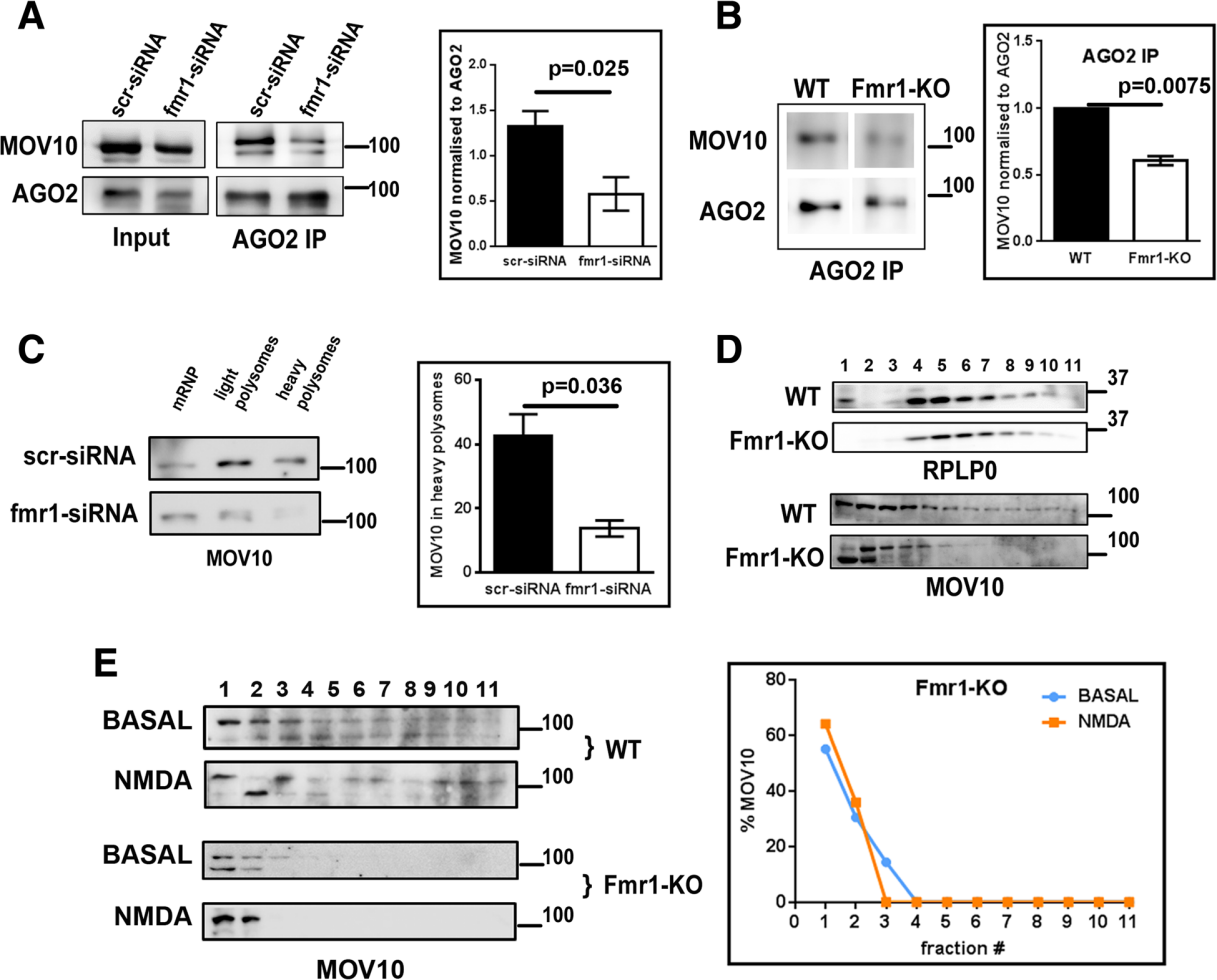
MOV10 interaction with AGO2 and its distribution in polysomes requires FMRP. **a** Immunoblots for MOV10 and AGO2 for input and after AGO2-immunoprecipitation on fmr1 knockdown in Neuro2a cells. Quantitative analysis of MOV10 association with AGO2 following AGO2 immunoprecipitation from Neuro 2a cells transfected with scramble siRNA (scr-siRNA) or fmr1 siRNA (fmr1-siRNA) (*n* = 4, unpaired Student’s t-test, ±SEM). **b** Immunoblots for MOV10 and AGO2 after AGO2-immunoprecipitation from wild type (WT) or Fmr1-KO synaptoneurosomes (WT and Fmr1-KO samples were run on separate immunoblots). Quantitative analysis of MOV10 association with AGO2 following AGO2 immunoprecipitation from WT or Fmr1-KO synaptoneurosomes (*n* = 3, unpaired Student’s t-test, ±SEM). **c** Immunoblots for MOV10 after sucrose step gradient on fmr1 knockdown in Neuro2a cells. Quantification of MOV10 in 30% fraction (heavy polysomes) separated on a sucrose step gradient from Neuro 2a cells transfected with scramble siRNA (scr-siRNA) or fmr1 siRNA (fmr1-siRNA). Values normalised to samples from scrambled siRNA transfected cells (n = 3, unpaired Student’s t-test, ±SEM). **d** Immunoblots of ribosomal protein RPLP0 and MOV10 on a linear sucrose gradient from wild-type (WT) or Fmr1 KO rat cortical synaptoneurosomes. **e** Immunoblots of MOV10 on a linear sucrose gradient from wild-type (WT) and Fmr1 KO rat cortical synaptoneurosomes on NMDAR stimulation. Distribution of MOV10 on linear sucrose gradient from Fmr1-KO rat synaptoneurosomes on NMDAR stimulation based on adjacent immunoblots

It is previously reported that MOV10 interacts with FMRP and AGO2 independently [26, 30]. Here we investigated the role of FMRP in AGO2-MOV10 interaction. Interestingly, when FMRP was knocked down in Neuro-2a cells by specific small interfering RNA (siRNA) against Fmr1 mRNA (Additional file 1: Figure S3A), MOV10 co-precipitation with AGO2 was significantly reduced (Fig. 2a) compared to the scramble siRNA treated cells. AGO2 levels did not show significant change on Fmr1 knockdown (Additional file 1: Figure S3B). To study the role of FMRP in the MOV10-AGO2 interaction in neurons, we used Fmr1-Knockout (Fmr1-KO) rat (Additional file 1: Figure S3C**)** synaptoneurosomes for AGO2 immunoprecipitation and polysome profiling assays. AGO2-MOV10 interaction was reduced in Fmr1-KO synaptoneurosomes compared to wild-type (WT) corroborating our Neuro 2a results (Fig. 2b). These results show that the absence of FMRP reduces the interaction of MOV10 with AGO2 both in Neuro 2a cells and in cortical synaptoneurosomes.

FMRP knockdown (Fmr1-siRNA) in Neuro 2a cells resulted in a significantly reduced association of MOV10 with polysomes (Fig. 2c). In the Fmr1-KO synaptoneurosomes, we could detect MOV10 only in the lighter fractions (fractions 1–5) of the linear sucrose gradient and absent in the polysomes (Fig. 2d) while there was no change in the distribution of ribosomes (based on RPLP0 western blot) (Fig. 2d)**.** Earlier we showed that when cortical synaptoneurosomes were stimulated with NMDA, there was a significant increase in the percentage of MOV10 in the heavy polysomes (Fig. 1e and f). This shift of MOV10 to polysomes on NMDAR stimulation was absent in the Fmr1-KO synaptoneurosomes (Fig. 2e)**.** Further, we also studied the role of AGO2 in the distribution of MOV10 in polysomes (Additional file 1**:** Figure S3D). In the absence of AGO2, the presence of MOV10 in polysomes was not affected (Additional file 1: Figures S3E). These results confirm that FMRP is not only required for the association of MOV10 with AGO2 and translating polysomes at basal state but also for the shift of MOV10 from AGO2 to polysomes in response to NMDAR stimulation.

### Translation of specific mRNAs is affected by the absence of MOV10 and FMRP

**Fig. 3:**
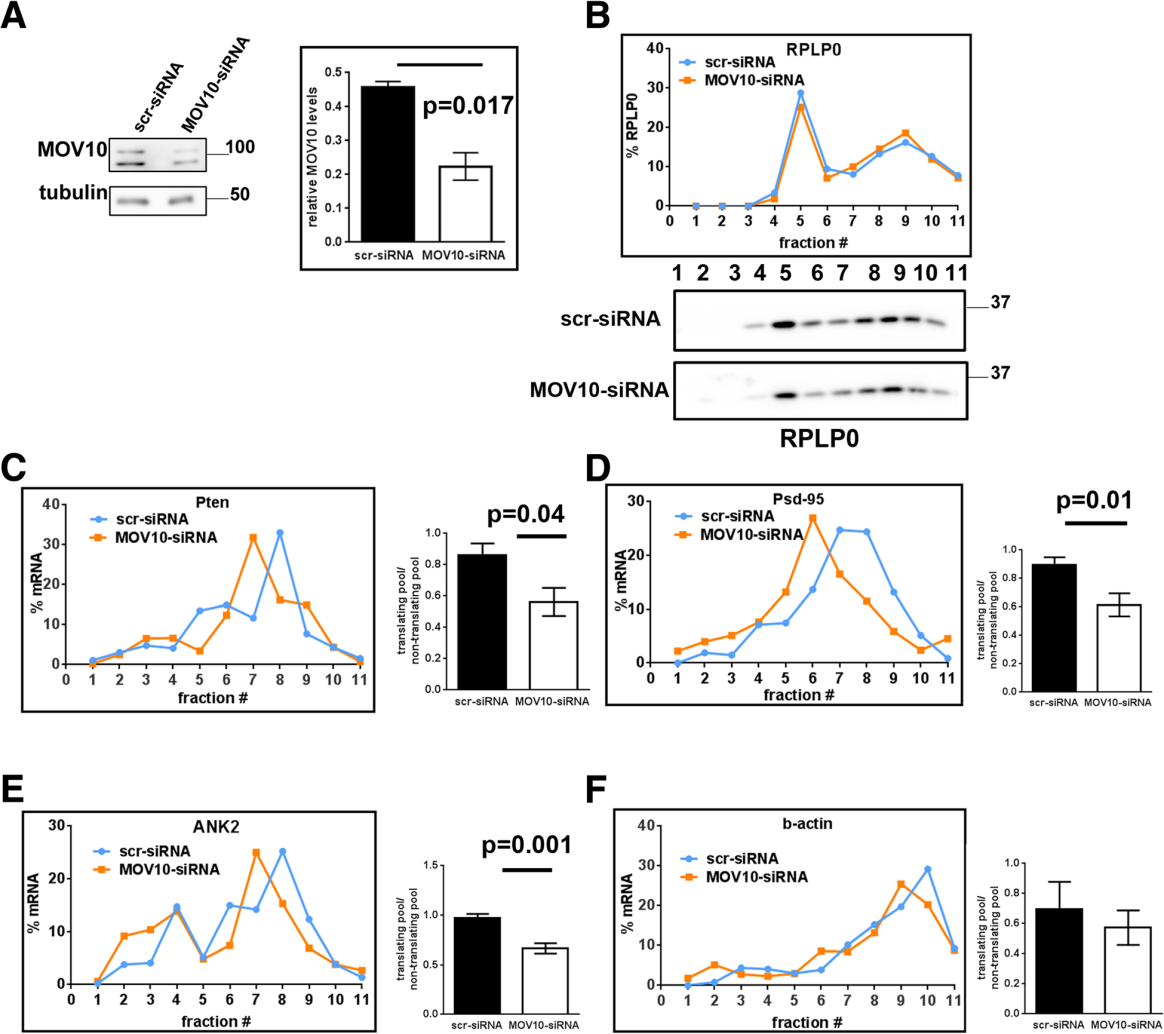
Translation of specific mRNAs is affected on MOV10 knockdown. **a** Immunoblots showing MOV10 knockdown from primary neuronal cultures transfected with scrambled siRNA (scr-siRNA) or MOV10 siRNA (MOV10-siRNA). Quantification of MOV10 in from primary neurons transfected with scramble siRNA (scr-siRNA) or MOV10 siRNA (MOV10-siRNA). Values normalised to samples from scrambled siRNA transfected cells (*n* = 3, unpaired Student’s t-test, ±SEM). **b** Distribution of RPLP0 on a linear sucrose gradient from primary neurons transfected with scrambled or MOV10 siRNA based on immunoblots shown below. **c**-**f** Distribution of selected mRNAs on linear sucrose gradient from primary neurons transfected with scrambled siRNA (scr-siRNA) or MOV10 siRNA (MOV10-siRNA) followed by quantification of mRNAs in polysomes (bar graphs, n = 4–6, unpaired Student’s t-test, ±SEM) for the mRNAs Pten (**c**), Psd-95 (**d**), Ank2 (**e**) and b-actin (**f**). Also see Additional file 1: Figure S4D-S4G

MOV10 is reported to interact with a large number of mRNAs [28, 31]. Since MOV10 is specifically dissociated from AGO2 on NMDAR stimulation and is shifted to the polysomal fraction (Fig. 1a, e), we investigated whether MOV10 has any effect on the translation of certain mRNAs. For this, we knocked down MOV10 (using siRNA) in primary neurons (Fig. 3a), and looked at the distribution of mRNAs in polysomes as compared to scrambled siRNA. Ribosomal protein RPLP0 did not show any change on MOV10 knockdown (Fig. 3b). Polysomal fractions were determined by the sensitivity to puromycin (fractions 8–11 were puromycin-sensitive in the case of primary neurons (Additional file 1: Figures S4A and S4B)). We chose mRNAs that were targets of MOV10 and/or FMRP from earlier reports [25, 26]. We saw a decrease in translation for the mRNAs phosphatase tensin homolog (Pten), Psd-95 and ankyrin 2 (Ank2) but no change for β-actin mRNA implying a role for MOV10 for these candidates (Fig. 3c-f and average line graphs in Additional file 1: Figures S4D-S4G). These candidates are also enriched in the pellet of MOV10 immunoprecipitation (Additional file 1**:** Figure S4C) as compared to that of Immunoglobulin G (IgG). These results indicate that MOV10 plays a role in translation activation of a specific set of mRNAs.

**Fig. 4:**
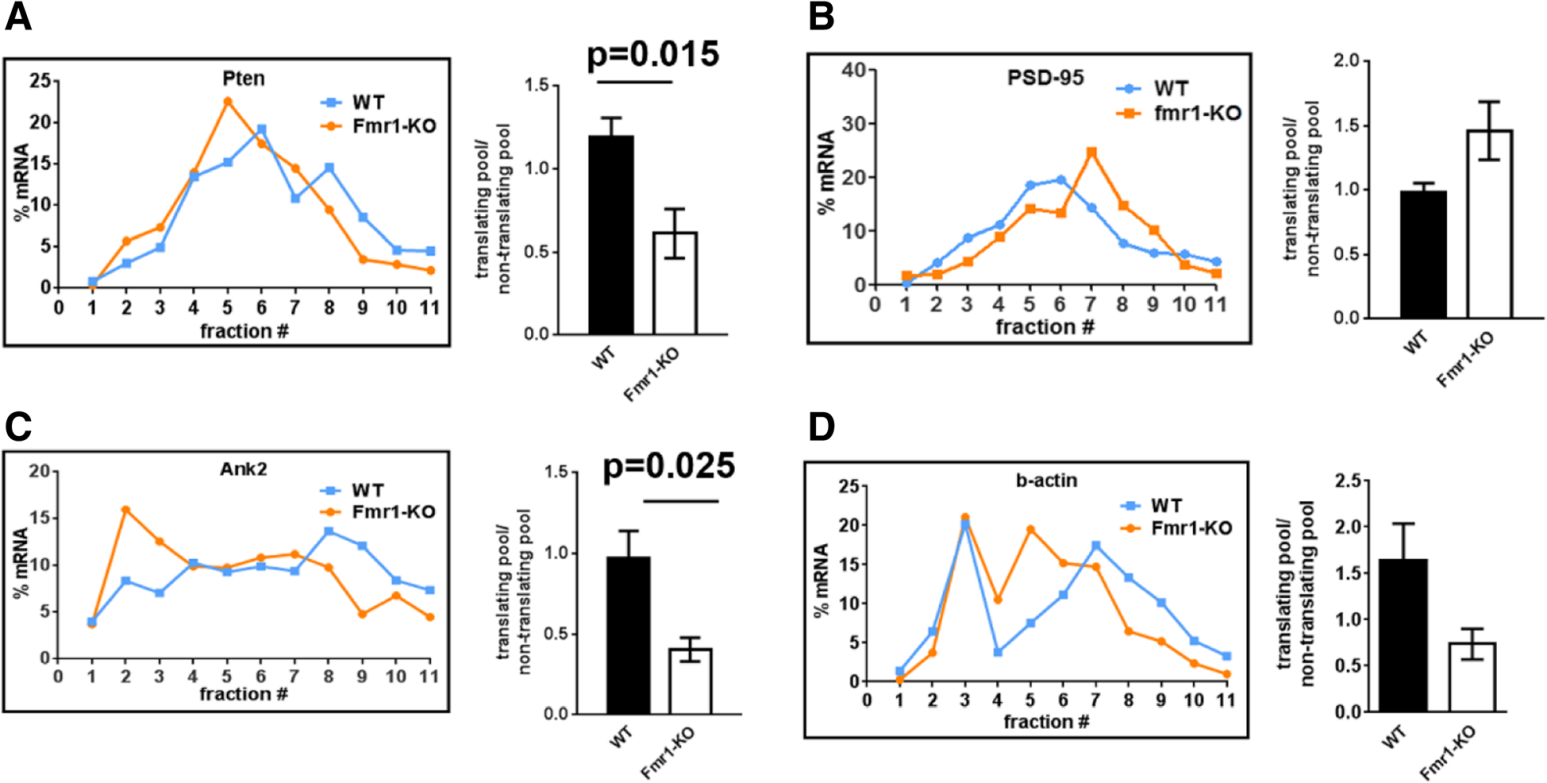
Translation of MOV10 target mRNAs is affected in Fmr1-KO synaptoneurosomes. **a**-**d** Distribution of selected (MOV10 targets) mRNAs on linear sucrose gradient from WT and Fmr1 KO rat synaptoneurosomes followed by quantification of mRNAs in polysomes (bar graph, n = 3–5, unpaired Student’s t-test ±SEM) for the mRNAs Pten (**a**), Psd-95 (**b**), Ank2 (**c**) and b-actin (**d**). Also see Additional file 1: Figure S5B-S5E

To study the role of FMRP in this context, we analysed the translation of MOV10 target mRNAs in Fmr1-KO synaptoneurosomes. Our selected MOV10 target mRNAs are also previously reported to be FMRP targets [25] and we further validated their association with FMRP (Additional file 1: Figure S5A) by immunoprecipitation. Interestingly, in the absence of FMRP, except for Psd-95 mRNA, both Pten and Ank2 mRNAs showed significant a reduction in translation as per their distribution in polysomal fractions (Fig. 4a and c, with average line graphs in Additional file 1: Figure S5B-S5E). The translation of Psd-95 mRNA increased in the absence of FMRP as previously shown [11] but this was not statistically significant (Fig. 4b)**.** β-actin mRNA also showed a trend of decrease on Fmr1-KO polysomes which was not statistically significant (Fig. 4d). These results indicate that the combination of FMRP and MOV10 have both distinct and convergent roles in the translation of mRNAs.

### NMDAR stimulation leads to translation of FMRP-MOV10 target mRNAs

**Fig. 5:**
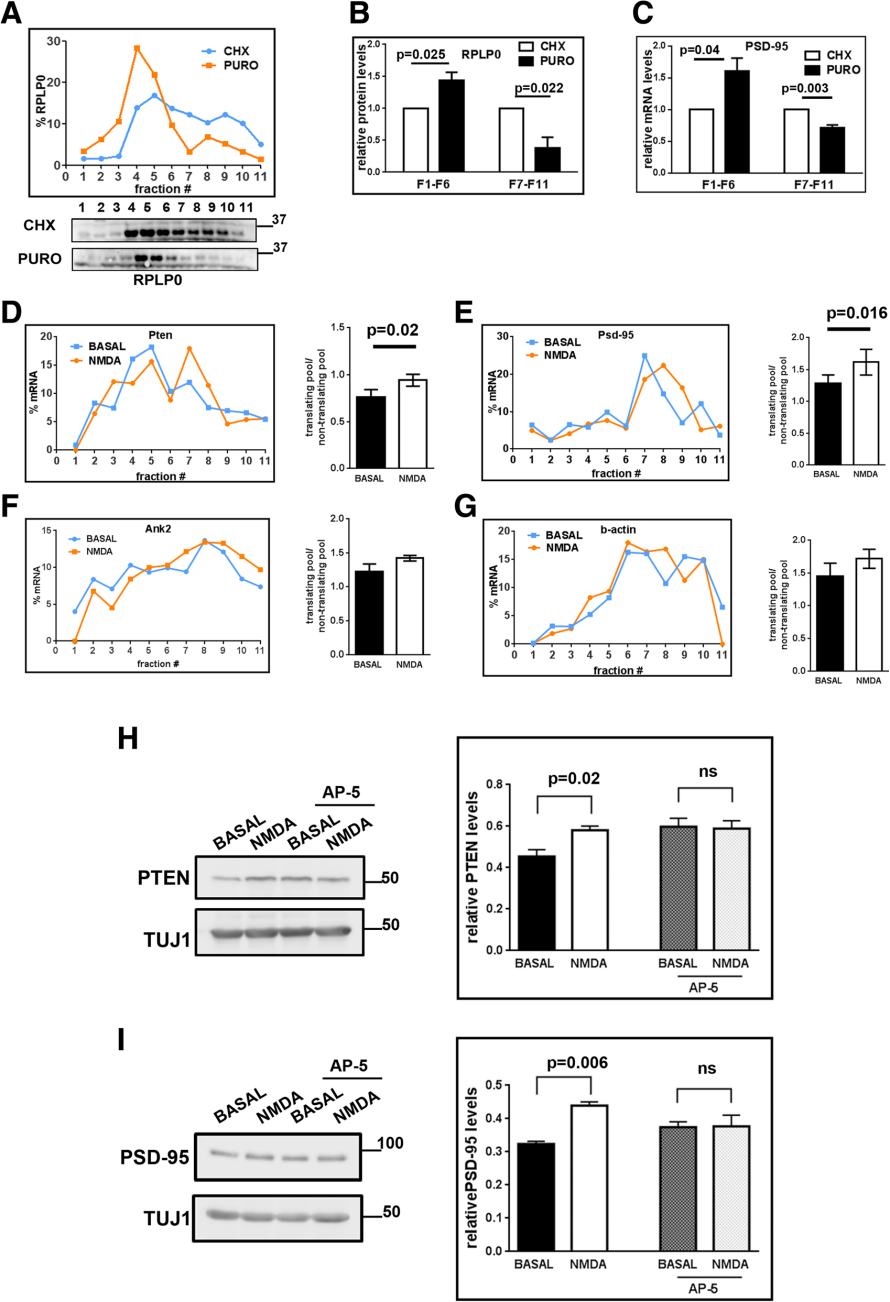
NMDAR stimulation leads to translational upregulation of MOV10-FMRP target mRNAs. **a** Ribosome distribution on linear sucrose gradient on cycloheximide and puromycin treatment based on RPLP0 immunoblot (below) from rat cortical synaptoneurosomes. **b**, **c** Quantitative distribution of RPLP0 protein or Psd-95 mRNA on linear sucrose gradient cycloheximide and puromycin treatment normalized to cycloheximide levels (n = 3, unpaired Student’s t test, ±SEM). **d**-**g** Distribution of mRNAs on linear sucrose gradient from synaptoneurosomes at basal state and on NMDAR stimulation followed by quantification of mRNAs in polysomes (bar graphs, n = 3, paired Student’s t-test, ±SEM) for the mRNAs; Pten (**d**), Psd-95 (**e**), Ank2 (**f**) and b-actin (**g**). Also see Additional file 1: Figure S6C-S6F. **h** Immunoblot showing PTEN protein after NMDAR stimulation with or without AP-5. Quantification of PTEN levels on NMDAR stimulation normalized to Tuj1 (*n* = 6, one way ANOVA, Tukey’s multiple comparison test, ±SEM). **i** Immunoblot showing PSD-95 protein after NMDAR stimulation with or without AP-5. Quantification of PSD-95 levels on NMDAR stimulation normalized to Tuj1 (n = 4, one way ANOVA, Tukey’s multiple comparison test, ±SEM)

On NMDAR stimulation, MOV10 moves into polysomes and absence of which leads to translation inhibition of specific mRNAs. To check whether these mRNAs undergo translation on NMDAR stimulation, we did polysome profiling from synaptoneurosomes after NMDAR stimulation. Translating polysomal fractions were determined by the sensitivity to puromycin (fractions 7–11 were puromycin-sensitive in the case of synaptoneurosomes (Fig. 5a) which is reflected by the decreased ribosomal protein RPLP0 from fraction 7–11 (Fig. 5a and b) and the corresponding increase in the early fractions). Similarly, the distribution of Psd-95 mRNA was also shifted out of fractions 7–11 in puromycin treated samples compared to cycloheximide-treated samples (Fig. 5c). Hence we considered the mRNAs present in fractions 7–11 as actively translating pool for further quantification, as also discussed previously [6, 32]. Next, we did polysome profiling from synaptoneurosomes after NMDAR stimulation. The profile for ribosomal protein RPLP0 did not significantly change for both basal and NMDAR stimulation conditions (Additional file 1: Figure S6A and S6B).

Among the four candidates that we tested in this assay, we found that Pten and Psd-95 mRNAs showed an increase in translating fractions on NMDAR stimulation and Ank2 and β-actin did not show any change (Fig. 5d-g, with average line graphs in Additional file 1: Figure S6C-S6F). Thus, NMDAR leads to translation activation of Pten and Psd-95 mRNAs which is mediated through both MOV10 and FMRP.

**Fig. 6:**
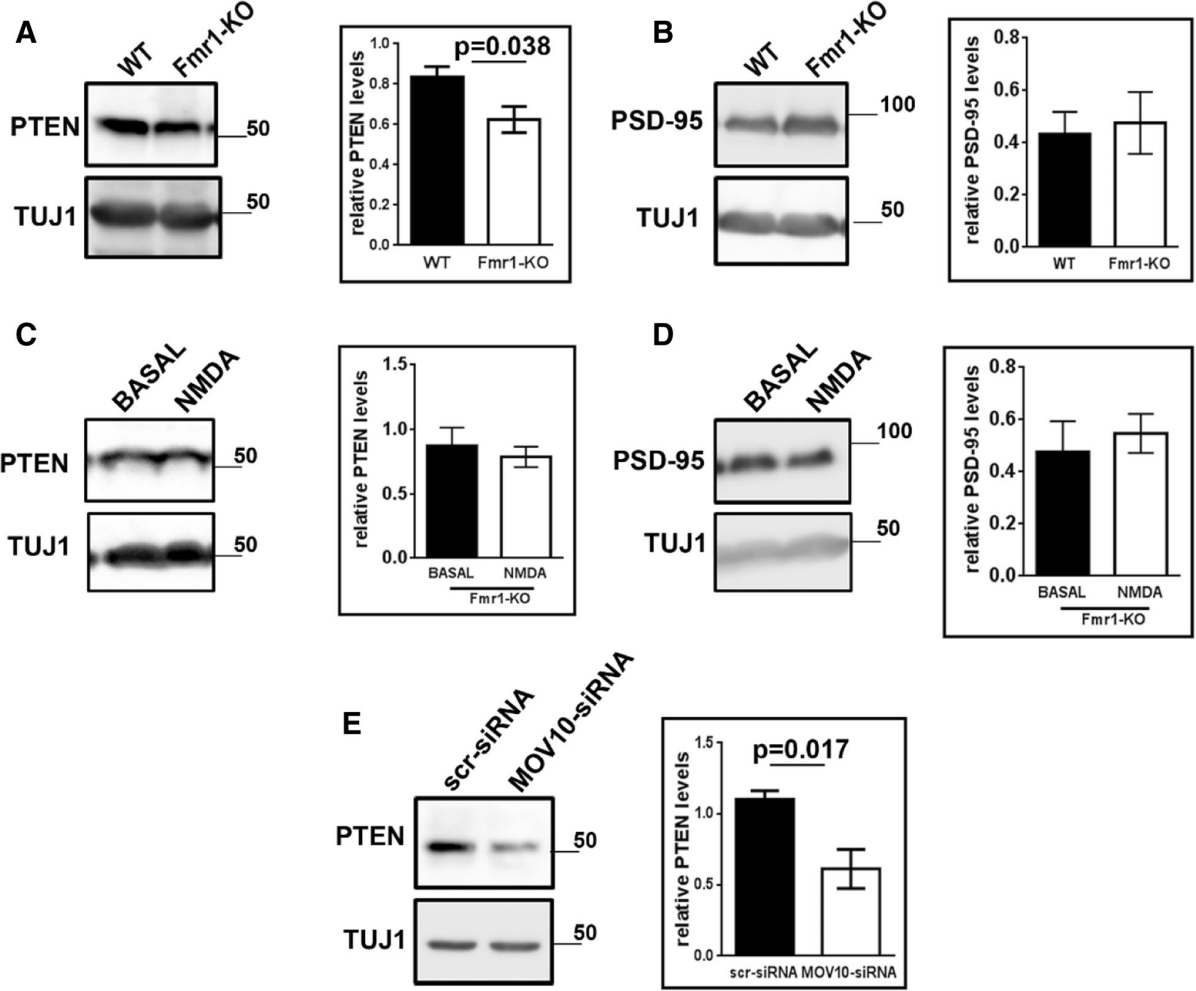
Pten and Psd-95 mRNAs are translationally regulated by FMRP-MOV10 on NMDAR stimulation. **a** Immunoblots showing PTEN protein from WT and Fmr1-KO synaptoneurosomes. Quantification of PTEN levels from WT and Fmr1-KO synaptoneurosomes normalized to Tuj1 (n = 5, unpaired Student’s t test, ±SEM). **b** Immunoblots showing PSD-95 protein from WT and Fmr1-KO synaptoneurosomes. Quantification of PSD-95 levels from WT and Fmr1-KO synaptoneurosomes normalized to Tuj1 (n = 3, unpaired Student’s t test, ±SEM). **c** Immunoblost showing PTEN protein after NMDAR stimulation from Fmr1-KO synaptoneurosomes. Quantification of PTEN levels on NMDAR stimulation normalized to Tuj1 (n = 3, paired Student’s t test, ±SEM). **d** Immunoblots showing PSD-95 protein after NMDAR stimulation from Fmr1-KO synaptoneurosomes. Quantification of PSD-95 levels on NMDAR stimulation normalized to Tuj1 (n = 3, paired Student’s t test, ±SEM). **e** Immunoblot showing PTEN protein from neurons transfected with scramble or MOV10 siRNA. Quantification of PTEN levels on MOV10 knockdown normalized to Tuj1 (n = 4, unpaired Student’s t test, ±SEM)

Among the mRNA candidates that we tested, we found Pten mRNA to be consistently significant for all the assays. To further validate this process, we looked at PTEN protein levels through western blotting (whole blots in Additional file 1**:** Figure S7A-S7B to show antibody specificity). Interestingly, we observed a significant increase in protein levels for both PTEN and PSD-95 on NMDAR stimulation in synaptoneurosomes as compared to basal conditions (Fig. 5h and i). This increase was lost when the stimulation was done in the presence of specific NMDAR inhibitor 2-Amino-5-Phosphonopentanoic acid (AP-5), confirming the role of NMDAR in translation upregulation of these mRNAs (Fig. 5h and i). We also observed a decrease in PTEN protein levels from Fmr1-KO synaptoneurosomes as compared to that in WT (Fig. 6a). PSD-95 protein levels showed an increase but were not significant from Fmr1-KO synaptoneurosomes (Fig. 6b) similar to the polysome profiling data (Fig. 4b). We also looked at the translation response to NMDAR stimulation in FMR1-KO synaptoneurosomes. We saw no change in both PTEN and PSD-95 protein levels on NMDAR stimulation in Fmr1-KO synaptoneurosomes (Fig. 6c and d), implying that FMRP is required for NMDAR mediated protein synthesis of Pten and Psd-95 mRNAs. In primary neurons, on knockdown of MOV10, we observed a decrease in PTEN protein levels, as compared to scrambled siRNA levels (Fig. 6e). Overall, these results confirm the translation activation of a specific subset of mRNAs on NMDAR stimulation mediated through MOV10 and FMRP.

### Dephosphorylated FMRP forms the inhibitory complex with MOV10-AGO2 and phosphorylation of FMRP dissociates this complex

**Fig. 7:**
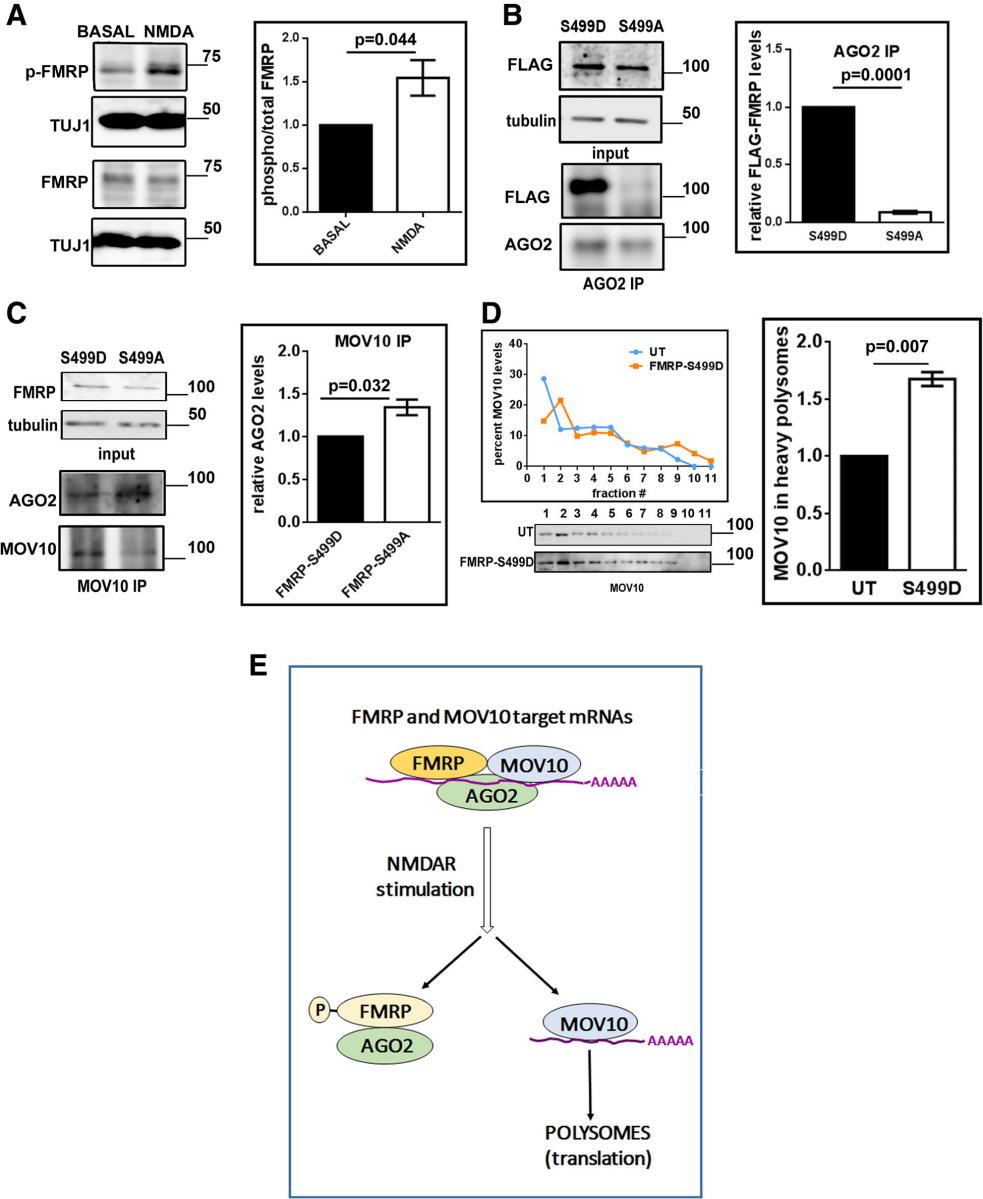
Phosphorylation of FMRP is the switch for NMDAR mediated translation. **a** Immunoblots for phospho FMRP (at S499) and total FMRP from synaptoneurosomes after NMDAR stimulation. Quantification of the ratio of phospho-FMRP to total-FMRP normalized to tuj1 for NMDAR stimulation (n = 6, paired Student’s t test, ±SEM).**b** Immunoblots for FLAG showing overexpression of FMRP-S499D, FMRP-S499A and tubulin from Neuro 2a cells. Bottom panel: Immunoblots for FLAG-FMRP and AGO2 after AGO2 immunoprecipitation from Neuro 2a cells transfected with FMRP-S499D or FMRP-S499A. Quantitative analysis of AGO2 association with FLAG-FMRP following AGO2 immunoprecipitation normalised to levels in the FMRP-S499D overexpressing cells. (n = 3, unpaired Student’s t-test, ±SEM). **c** Immunoblots for FMRP showing overexpression of FMRP-S499D, FMRP-S499A and tubulin from Neuro 2a cells. Bottom panel: Immunoblots for AGO2 and MOV10 after MOV10 immunoprecipitation from Neuro 2a cells transfected with FMRP-S499D or FMRP-S499A. Quantitative analysis of AGO2 association with MOV10 following MOV10 immunoprecipitation normalised to levels in the FMRP-S499D overexpressing cells. (n = 3, unpaired Student’s t-test, ±SEM). **d** Distribution of MOV10 separated on a linear sucrose gradient from Neuro 2a cells untransfected (UT) or transfected with FMRP-S499D based on immunoblots shown below followed by quantification of MOV10 in polysomes (n = 3, unpaired Student’s t-test, ±SEM). **e** Model illustrating the role of MOV10 in response to NMDAR stimulation which is mediated by FMRP and its phosphorylation status

Previously, it has been shown that the FMRP gets dephosphorylated downstream of mGluR signaling [11, 33]. In this study, we wanted to investigate the role of the phosphorylation state of FMRP in NMDAR mediated translation. For this, we quantitated the change in phosphorylation status of FMRP on NMDAR stimulation from cortical synaptoneurosomes. For this we used the antibody which specifically recognises the phosphorylated form of FMRP at S499 (Fig. 7a and Additional file 1: Figure S8A). On NMDAR stimulation there was a significant increase in phosphorylation of FMRP (Fig. 7a) with no change in total FMRP levels **(**Additional file 1: Figure S8B). To study the effect of this result on MOV10-FMRP-AGO2 interaction, we overexpressed phospho-mimetic or dephospho-mimetic form of FMRP (FMRP-S499D and FMRP-S499A respectively), in Neuro 2a cells. In this condition, as previously reported [11] AGO2 interacts more with phospho-mimetic form of FMRP (FMRP-S499D) compared to dephospho-mimetic form (FMRP-S499A) (Fig. 7b and Additional file 1: Figure S8C2). We did MOV10 immunoprecipitation in the overexpression background and quantitated the amount of AGO2 co-precipitated with MOV10. Here we observed increased AGO2 co-precipitation with MOV10 from the cells overexpressing FMRP-S499A (dephosphorylated FMRP mimetic) compared to the cells overexpressing FMRP-S499D (phosphorylated FMRP mimetic) (Fig. 7c and Additional file 1: Figure S8C1). Thus, phosphorylated FMRP appears to promote the dissolution of AGO2-MOV10 inhibitory complex which is in contrast to the previous result that phosphorylated FMRP promotes FMRP-AGO2 inhibitory complex [11]. Polysome profiling from Neuro 2a cells overexpressing FMRP-S499D led to an increase in the percentage of MOV10 in polysomes (Fig. 7d and Additional file 1: Figure S8D) as compared to un-transfected cells indicating that overexpression of FMRP-S499D shifts MOV10 to translating polysomes. On the other hand overexpression of FMRP-S499A led to a decrease of MOV10 in polysomes (Additional file 1: Figure S8E). Overexpression of FMRP-S499D or FMRP-S499A did not affect the overall polysome profile based on RPLP0 western blot (Additional file 1: Figure S8E) but has a significant impact on the distribution of MOV10 in polysomes. Thus, these results indicate that phosphorylation of FMRP is likely the switch downstream of NMDAR that shifts MOV10 to polysomes and promotes translation of its target mRNAs.

## Discussion

Protein synthesis is known to play an important role downstream of both NMDAR and mGluR stimulation [34]. But currently, there is no clear understanding regarding the distinct translational response downstream of these pathways. Though mGluR stimulation is correlated with global translation activation and NMDAR stimulation with translation inhibition, there are many contrasting reports when it comes to individual transcripts [9, 12, 35].

Since there is an overlap of many signalling components between NMDAR and mGluR [34], we hypothesized that similar to mGluR mediated translation regulation, specificity of NMDAR mediated translation is regulated at the messenger ribonuclearprotein (mRNP)-mRNA level [11]. RNA binding proteins such as FMRP, Human antigen R (HuR), Staufen2, and MOV10 play a crucial role in regulating translation of target mRNAs in a reversible manner. Staufen2 (Stau 2) is required for the transport and translation of microtubule associated protein 1b (Map1b) mRNA downstream of mGluR activation [36] whereas interaction of HuR with cationic amino acid transporter (CAT-1) mRNA was shown to relieve it from miRISC mediated inhibition in response to stress [20]. MOV10 is the mammalian homolog of Drosophila Armitage protein which is shown co-localize with AGO2 in HEK cells and is a component of miRISC [30]. An earlier study in hippocampal neurons has linked MOV10 to NMDAR mediated translation activation [19]. MOV10 is known to bind to mRNAs [31] and regulate the translation of CamK2a, lysophospholipase 1 (lypla1) mRNAs via their 3′ untranslated region (3’UTRs) [19]. In the above studies, the RNA binding proteins are shown to influence microRNA mediated inhibition and the translation of specific mRNA in response to particular signalling cues. In this regard, MOV10 was an ideal candidate for NMDAR mediated translation regulation as its role has been established previously [19, 27]. In order to characterise its regulatory role, we looked at MOV10 association with miRISC protein AGO2 and polysomes in synaptoneurosomes.

We found that MOV10 associates with both miRISC (AGO2) and polysomes and thus is involved in both translation inhibition and activation. On NMDAR signaling, the association of MOV10 with AGO2 decreases and it concomitantly increases in translating polysomes. Thus, MOV10 promotes the active translation of its bound mRNAs on NMDAR stimulation. In agreement with this, MOV10 knockdown showed a decrease in translation of its selected target mRNAs shown by polysome profiling. These results indicate that MOV10 not only acts an inhibitory RBP as shown previously [19, 27] but also has a role in translation activation of mRNAs downstream of NMDAR stimulation.

We also show that another RBP, FMRP, has a crucial role in MOV10 mediated translation regulation downstream of NMDAR activation. The role of FMRP as a translational regulator is well established in response to mGluR stimulation [24], but very little is known about the role of FMRP in the context of NMDAR stimulation. In this study, we show that there is an active translation of a specific subset of mRNAs downstream of NMDAR stimulation and FMRP along with MOV10 is critical for this regulation. Our data shows that FMRP is essential for the formation of MOV10-AGO2 inhibitory complex and for the shift of MOV10 (along with its target mRNAs) to translating polysomes on NMDAR stimulation. Studying the phosphorylation status of FMRP seems to provide the key molecular insight in understanding synaptic translation. In contrast to mGluR stimulation as shown previously [11], we found that on NMDAR stimulation there is an increase in the phosphorylation of FMRP at S499. Overexpression of phospho-mimetic of FMRP (FMRP-S499D) increases the FMRP-AGO2 complex but leads to the shift of MOV10 from AGO2 to translating polysomes. In contrast, dephospho-mimetic FMRP (FMRP-S499A) leads to an increase in MOV10-FMRP-AGO2 complex and decreased the MOV10 in translating polysomes. Based on these data, we propose a model (Fig. 7e) that indicates a possible mechanism for NMDAR mediated translation activation through FMRP and MOV10. These results indicate that NMDAR mediated FMRP phosphorylation has an effect on MOV10 mediated translation of mRNAs. Phosphorylation of FMRP is the switch downstream of NMDAR that leads to MOV10 moving into polysomes promoting translation of its target mRNAs.

We were also able to show that translation of Pten mRNA is upregulated on NMDAR activation and is regulated by FMRP and MOV10. PTEN is a known inhibitor of Protein Kinase B (Akt/PKB) pathway and pten mutations have been linked with autism spectrum disorders (ASD) [37]. We have used polysome profiling and immunoprecipitation along with MOV10 knockdown and FMRP knockout systems to show their role in NMDAR mediated translation but there is a scope to further test the roles of these RBPs in NMDAR signalling. Thus, in summary, this work draws attention to the importance of studying the role of FMRP beyond mGluR stimulation and particularly in NMDAR mediated signalling which will have a clear bearing on the molecular pathology of Fragile X Syndrome (FXS) and autism spectrum disorders (ASD).

## Materials and methods

**Cell line and primary neuronal culture:** Primary neuronal cultures were prepared from cerebral cortices of embryonic day 18 (E18) rats (Sprague-Dawley) according to the established protocol [38]. 2-3 × 10^6^ dissociated cells were plated on poly-L-lysine (0.2 mg/ml in borate buffer, pH 8.5) coated 10 cm culture dishes. Neurons were attached to the substrate in minimal essential medium with FBS (10%) for 3 h, then later grown in defined Neurobasal Medium (Invitrogen) with GlutaMAX™ supplement (Gibco™) and B-27 supplements (Invitrogen). Neurons were cultured for 14d at 37 °C in a 5% CO_2_ environment. For knockdown studies in neurons, NeuroMag (OZ Biosciences) was used as the transfection reagent. Silencer select siRNAs from Ambion against MOV10 transcript were transfected on days in vitro (DIV) 12 and the neurons were lysed on DIV 14.

Neuro2a cells were maintained in DMEM (Gibco®) with 10% FBS (Sigma) and GlutaMAX™ supplement (Gibco™). For knockdown studies, Silencer Select siRNA from Ambion were used. siRNA transfections were done using Lipofectamine® 2000 transfection reagent. For overexpression studies, phosphomutants of FMRP, FMRP-S499D and FMRP-S499A plasmid constructs [11] were transfected using Lipofectamine® 2000 and cells were lysed 24 h after the transfection.

### Immunoprecipitation

Immunoprecipitation was done using anti-EiF2C2 (Abnova H00027161-MO1), anti-FMRP (Sigma-F4055), anti-MOV10 (Abcam-ab80613), Mouse IgG (Abcam-ab37355) and protein G Dyna beads (Invitrogen). Samples were processed for either western blotting or quantitative (real-time) PCR following immunoprecipitation as described previously [11]. The above antibodies including RPLP0 (Abcam-ab101279), α-tubulin (Sigma T9026), FLAG M2 (Sigma Millipore F3165) and β-III tubulin (Tuj1, Sigma T8578), phospho FMRP-S499 (Abcam-ab183319) were used for immunoblotting.

### Sucrose step gradient

800 μl of 20% sucrose solution was overlaid on 800 μl of 30% sucrose solution. 400 μl cell lysate was added and the centrifugation was carried out at 40,200 rpm for 2 h in SW 50.1 rotor (Beckman Coulter) [29]. Fractions were then collected and analyzed by qPCR and western blotting. All sucrose solutions were made in gradient buffer (20 mM Tris-Cl pH 7.4, 100 mM KCl, 5 mM MgCl_2_ 0.1 mg/ml cycloheximide, protease inhibitor and RNase inhibitor). The lysis buffer consisted of the gradient buffer with 1% Nonidet P-40 (NP40). For puromycin treatment, 1 mM puromycin was added to Neuro 2a cells or synaptoneurosomes and incubated for 2 h or 30 min respectively at 37 °C before lysis.

### Synaptoneurosome preparation

Cortical synaptoneurosomes were prepared by differential filtration method [6] from Sprague Dawley (SD) WT or fmr1 KO [39] rats. For stimulation, synaptoneurosome solution was pre-warmed at 37 °C for 5 min and then stimulated with N- Methyl-D-Aspartate (NMDA, Sigma 20 μM) for 5 min at 37 °C with mock stimulation considered as the basal condition.

For PTEN and PSD-95 protein detection, post NMDAR stimulation, the synaptoneurosomes were pelleted, the buffer was replaced with fresh synaptoneurosomes buffer and the synaptoneurosomes were incubated at 37 °C for additional 20 min. Synaptoneurosomes were then lysed and denatured by SDS-denaturing buffer. For AP-5 treatment, the synaptoneurosomes were pre-incubated with AP-5 (100 μM) for 10 min at 37 °C, stimulated with NMDA for 5 min at 37 °C. Post NMDAR stimulation, the synaptoneurosomes were further incubated at 37 °C for 20 min in fresh synaptoneurosome buffer and then lysed and denatured by SDS-denaturing buffer. Anti-PTEN (CST 9552S) and anti-PSD-95 (Abcam 76,115) antibodies were used for western blotting.

### Electron microscopy

Electron microscopy was done from synaptoneurosomes as described earlier [40]. Synaptoneurosomes were fixed in 4% paraformaldehyde and 2.5% glutaraldehyde in 0.1M sodium cacodylate. Fixed samples after washes were then embedded in epoxy resin at 60C for 48h. Blocks were sectioned and imaged using TEM (FEI-Technai biotwin T12) at 100kV.

### Polysome profiling

Polysome assay was done from synaptoneurosome samples after stimulation [6]. In brief, synaptoneurosome/cell lysate was separated on a 15–45% linear sucrose gradient in the presence of cycloheximide or puromycin. 1.0 mL fractions were collected and used for further analysis through western blot and qPCR.

Quantitative analysis for polysome profiling: qPCR data was analysed by the absolute quantification method using a standard curve as mentioned previously [41]. Absolute copy numbers for a particular mRNA were obtained from each of the 11 fractions. These copy numbers were then represented as percentage distribution across the 11 fractions.

#### For synaptoneurosomes

Fractions 7 to 11 were considered as translating pool based on sensitivity to puromycin (Fig. 5a).

Translating pool**/**non-translating pool = sum of the percentage of mRNA from fraction 7 to fraction 11 **÷** sum of the percentage of mRNA from fraction 1 to fraction 6.

#### For primary neurons (Additional file 1: Figure S3A)

Translating pool**/**non-translating pool = sum of the percentage of mRNA from fraction 8 to fraction11 **÷** sum of the percentage of mRNA from fraction 1 to fraction 7.

### Quantitative PCR primers

For 18S ribosomal RNA (rRNA) quantification, cDNA samples were diluted one thousand times and then used for qPCR.

List of Primers

**Table:** 

Transcript	Forward sequence (5’→3′)	Reverse Sequence (5’→3′)
Psd-95	ATGGCAGGTTGCAGATTGGA	GGTTGTGATGTCTGGGGGAG
18S rRNA	GGTGACGGGGAATCAGGGTTCGAT	TTTTCGTCACTACCTCCCCGGGTC
Pten	AGGACCAGAGATAAAAAGGGAGT	CCTTTAGCTGGCAGACCACA
Ankyrin2	ACCCTGCCAATTTATGCCAAG	GTTTCTGTCGACTCTGTCTCA
β-actin	GGCTCCTAGCACCATGAAGAT	AAACGCAGCTCAGTAACAGTC

### Statistical analysis

Group comparisons were made using one way analysis of variance (ANOVA) followed by Tukey’s multiple comparison test. Statistical significance was calculated using paired/unpaired Student’s t-test for biochemical experiments as mentioned. Data are presented as mean ± Standard error of Mean (SEM). *p* values less than 0.05 were considered statistically significant.

## Additional file

Additional file 1: **Figure S1.** Effect of NMDAR stimulation on MOV10 interaction with AGO2 (related to Fig. 1). **Figure S2.** Sucrose step gradient to isolate mRNPs, light and heavy polysomes (related to Fig. 1). **Figure S3.** FMRP regulates translation of NMDAR target mRNAs through MOV10 (related to Fig. 2). **Figure S4.** Puromycin sensitive fractions in primary neurons and validation of MOV10 targets by RNA-IP and (related to Fig. 3). **Figure S5.** Validation of FMRP targets by RNA-IP (related to Fig. 4). **Figure S6.** NMDAR stimulation leads to no change in the RPL0 distribution (related to Fig. 5). **Figure S7.** Whole blots for PTEN and PSD-95 to show antibody specificity (related to Fig. 6). **Figure S8.** Phosphorylation of FMRP is the switch for NMDAR mediated translation (Related to Fig. 7). (DOCX 3830 kb)

## Abbreviations

AGO2: Argonaute 2
Ank2: Ankyrin 2
ANOVA: Analysis Of Variance
AP-5: 2-Amino-5-Phosphonopentanoic acid
Arc: Activity regulated cytoskeleton associated protein
ASD: Autism Spectrum Disorder
Camk2a: Calcium/calmodulin dependent kinase II alpha
CHX: cycloheximide
DIV: days in vitro
E18: Embryonic day 18
Fmr1: fragile X mental retardation 1
FMRP: Fragile X Mental Retardation Protein
FXS: Fragile X Syndrome
Grin2a: Glutamate receptor ionic epsilon 1
HuR: Human antigen R
IgG: Immunoglobulin G
IP: Immunoprecipitation
KO: Knockout
lypla1: lysophospholipase 1
mGluR: metabotropic Glutamate Receptor
miRISC: miRNA Induced Silencing Complex
MOV10: Moloney Leukemia Virus 10
mRNP: messenger ribonucleprotein
NMDAR: N-Methly-D-Aspartate Receptor
NP40: Nonidet P-40
P30: postnatal day 30
PKB: Protein Kinase B
PSD: Postsynaptic density
Psd-95: postsynaptic density 95
Pten: phosphatase tensin homolog
PURO: Puromycin
qPCR: quantitative PCR
RBP: RNA Binding Protein
RPLP0: Ribosomal Protein Lateral stalk subunit P0
rRNA: ribosomal RNA
SD: Sprague Dawley
siRNA: small interfering RNA
Stau2: Staufen 2
SV: Synaptic Vesicles
UTR: Untranslated region
WT: Wild-Type
