# Supplementary material for: NMDAR mediated translation at the synapse is regulated by MOV10 and FMRP
Source: Mol Brain. 2019 Jul 10;12:65. doi: 10.1186/s13041-019-0473-0 (PMC6617594; doi:10.1186/s13041-019-0473-0)
Supplement: Supplementary file 1 — Figure S1. Effect of NMDAR stimulation on MOV10 interaction with AGO2 (related to Fig. 1). Figure S2. Sucrose step gradient to isolate mRNPs, light and heavy polysomes (related to Fig. 1). Figure S3. FMRP regulates translation of NMDAR target mRNAs through MOV10 (related to Fig. 2). Figure S4. Puromycin sensitive fractions in primary neurons and validation of MOV10 targets by RNA-IP and (related to Fig. 3). Figure S5. Validation of FMRP targets by RNA-IP (related to Fig. 4). Figure S6. NMDAR stimulation leads to no change in the RPL0 distribution (related to Fig. 5). Figure S7. Whole blots for PTEN and PSD-95 to show antibody specificity (related to Fig. 6). Figure S8. Phosphorylation of FMRP is the switch for NMDAR mediated translation (Related to Fig. 7). (DOCX 3830 kb) [file 13041_2019_473_MOESM1_ESM.docx]

**Supplementary Info for:**

**NMDAR mediated translation at the synapse is regulated by MOV10 and FMRP**

Preeti Madhav Kute^1, 2^, Sarayu Ramakrishna^1, 4^, Nagammal Neelagandan^1^, Sumantra Chattarji^1, 3,5^, Ravi S. Muddashetty^1,#,*^

^1^Centre for Brain development and Repair (CBDR), Institute for Stem Cell Biology and regenerative medicine (inStem), Bangalore-560065, India

^2^Shanmugha Arts, Science, Technology & Research Academy (SASTRA) University, Thanjavur-613401, India

^3^National Centre for Biological Sciences (NCBS), Bangalore-560065, India

^4^The University of Trans-Disciplinary Health Sciences and Technology, Bangalore-560064, India

^5^Centre for Integrative Physiology, Deanery of Biomedical Sciences, University of Edinburgh, Edinburgh, United Kingdom.

Preeti Madhav Kute- preetimk@instem.res.in, Sarayu Ramakrishna- sarayur@instem.res.in, Nagammal Neelagandan- nagammaln10@gmail.com, Sumantra Chattarji- shona@ncbs.res.in, Ravi S. Muddashetty- ravism@instem.res.in

^#^ Lead Contact, *Correspondence

**Running title:** Synaptic translation regulation by FMRP-MOV10


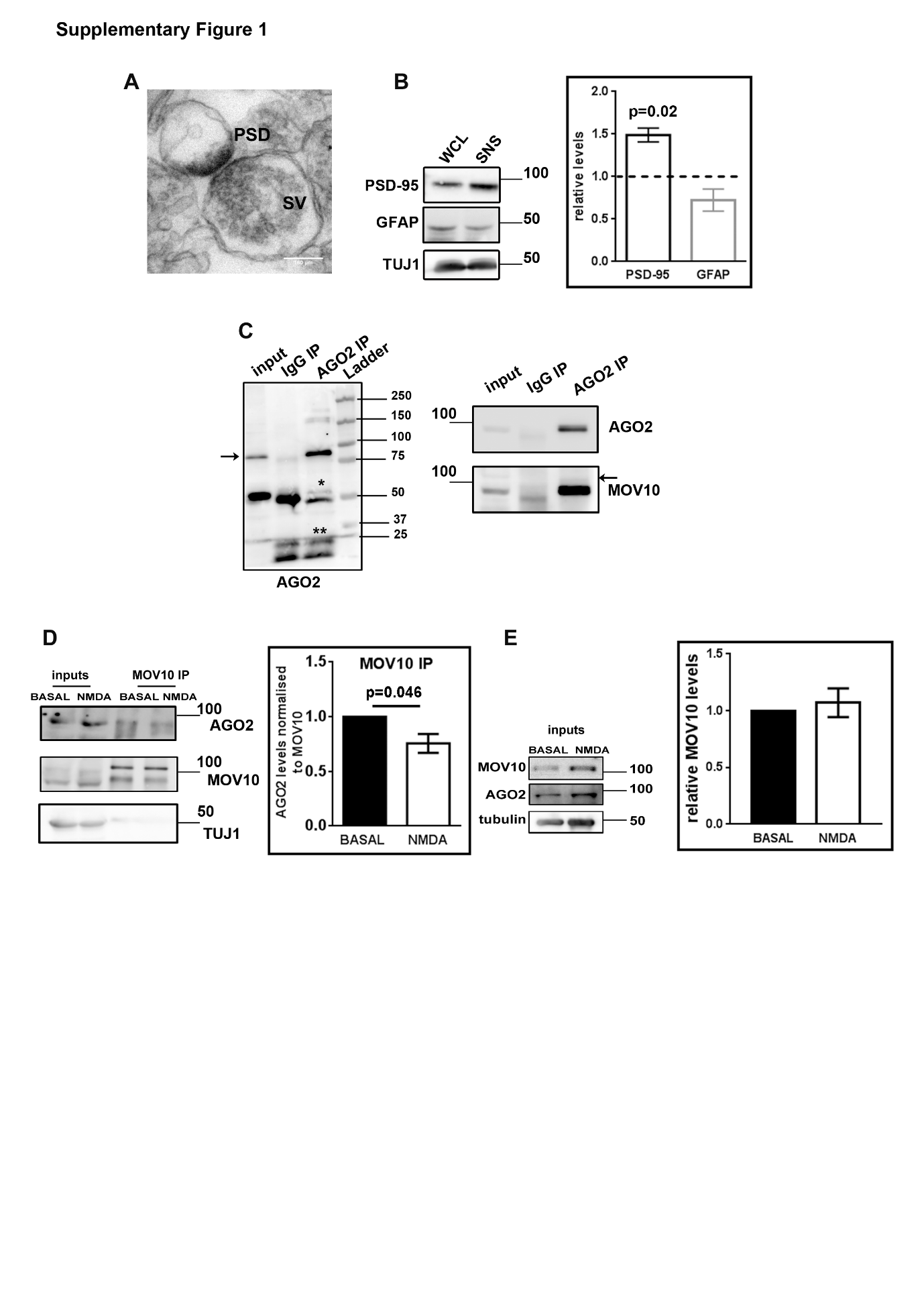


**Supplementary Figure 1:** Effect of NMDAR stimulation on MOV10 interaction with AGO2 (related to Figure 1)

1. Electron micrograph of representative sample of synaptoneurosomes isolated from rat cortex. Image shows sealed pre synaptic compartments with neurotransmitter vesicles or synaptic vesicles (SV) and post synaptic compartments with post synaptic density (PSD).
2. Post synaptic protein PSD-95 enrichment and glial protein GFAP de-enrichment in synaptoneurosomes as compared to whole cell lysate (WCL). Tuj1 is used as a loading control. Quantitative analysis of PSD-95 and GFAP levels as compared to Tuj1 from synaptoneurosomes, further normalized to WCL levels (dotted line) (n=3, unpaired t test with respect to WCL, ±SEM)
3. Representative blot for AGO2 IP, probed with anti-AGO2 antibody from rat synaptoneurosomes. Arrow denotes AGO2 band at ~95kDa, asterisk indicates denatured heavy chain (*) and light chain (**) of IgG. Adjacent immunoblot for AGO2 for input and after IgG or AGO2 immunoprecipitation from rat synaptoneurosomes. The same blot was reprobed for MOV10 where the MOV10 band observed above the AGO2 band in input lane and a faint band is seen for AGO2 IP lane (shown by arrow) which is absent in IgG IP lane.
4. Immunoblots for MOV10 and AGO2 for inputs and after MOV10-immunoprecipitation on NMDAR stimulation. Quantitative analysis of AGO2 association with MOV10 following MOV10 immunoprecipitation on NMDAR stimulation in rat cortical synaptoneurosomes (n=5, paired Student’s t-test, ±SEM). Values are normalized to basal levels.
5. Immunoblots for MOV10 and AGO2 for inputs and after AGO2-immunoprecipitation on NMDAR stimulation. Quantitative analysis of MOV10 on NMDAR stimulation in rat cortical synaptoneurosomes (n=3, ±SEM). Values are normalized to basal levels.


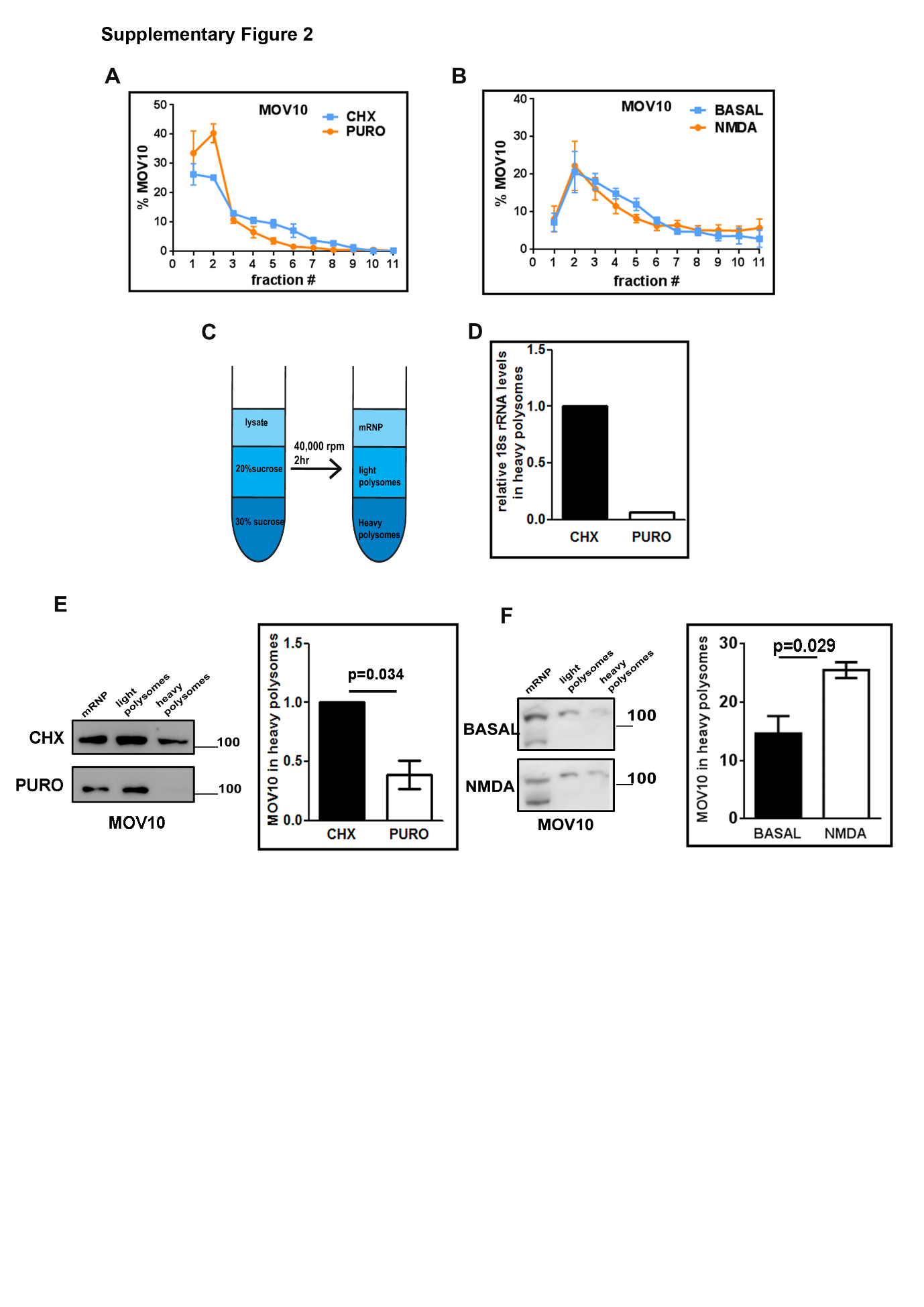


**Supplementary Figure 2:** Sucrose step gradient to isolate mRNPs, light and heavy polysomes (related to Figure 1)

1. Average distribution of MOV10 on linear sucrose gradient from rat cortical synaptoneurosomes after cycloheximide or puromycin treatment (n=3, ±SEM).
2. Average distribution of MOV10 on linear sucrose gradient from rat cortical synaptoneurosomes after NMDAR stimulation (n=5, ±SEM).
3. Schematic of sucrose step gradient to differentially sediment mRNPs, light polysomes, and heavy polysomes.
4. Quantitative analysis for 18S rRNA in heavy polysomes separated on a sucrose step gradient after cycloheximide (CHX) and puromycin (PURO) in neuro 2a cells. Values are normalized to cycloheximide levels.
5. Quantitative analysis for MOV10 in heavy polysomes on a sucrose step gradient after cycloheximide (CHX) and puromycin (PURO) in neuro 2a cells (n=3, unpaired Student’s t-test, ±SEM). Values are normalized to cycloheximide levels.
6. Quantitative analysis for MOV10 in heavy polysomes on a sucrose step gradient after NMDA stimulation in synaptoneurosomes (n=4, paired Student’s t-test, ±SEM).


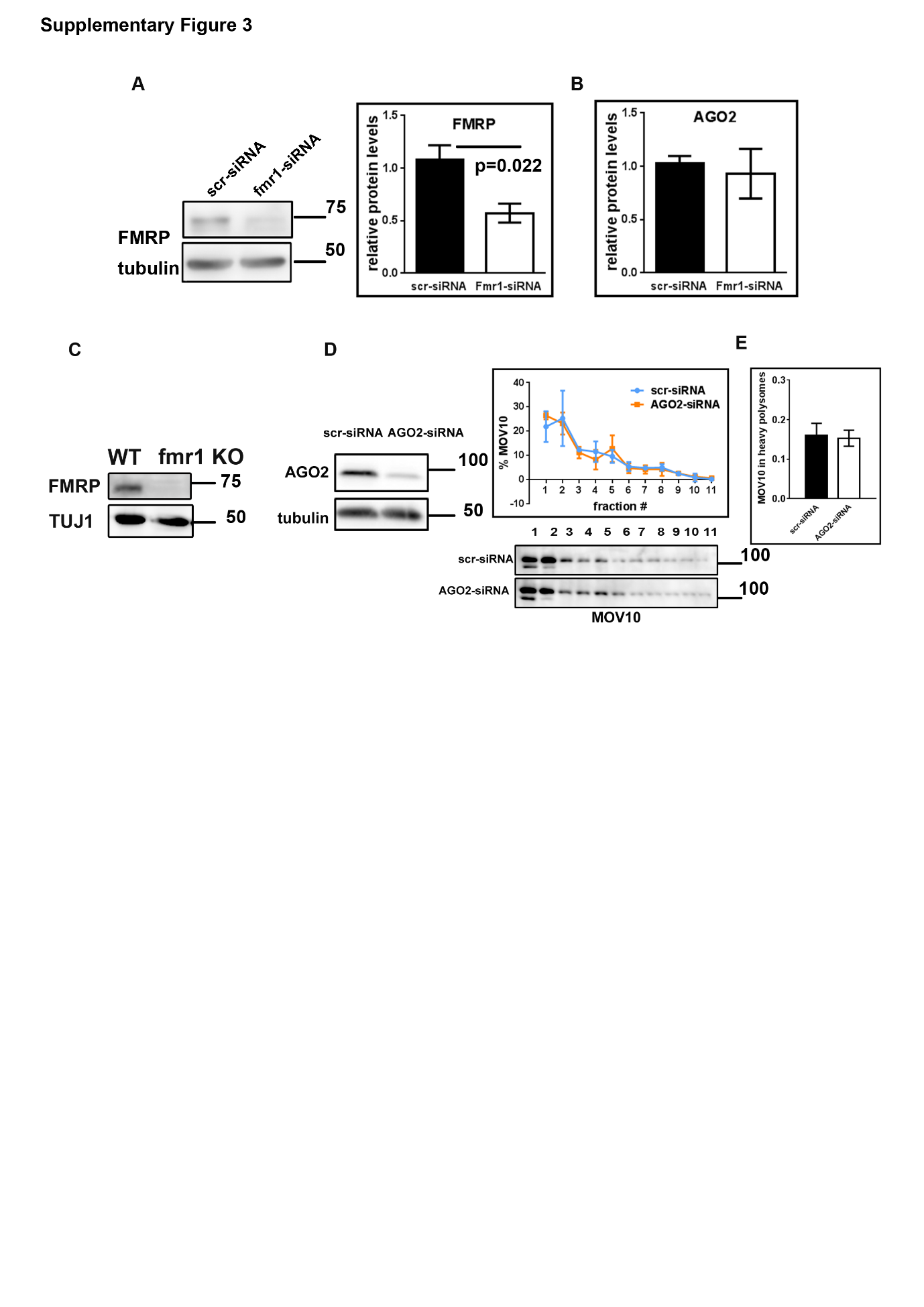


**Supplementary Figure 3**: FMRP regulates translation of NMDAR target mRNAs through MOV10 (related to Figure 2)

1. Immunoblots for FMRP and tubulin from neuro-2a cells transfected with scrambled siRNA (scr-siRNA) or fmr1 siRNA (fmr1-siRNA). Quantification of FMRP knockdown in neuro2a cells transfected with scrambled siRNA (scr-siRNA) or fmr1 siRNA (fmr1-siRNA). Values are normalized to FMRP levels in scrambled siRNA (n=3, unpaired Student’s test, ±SEM).
2. Quantification of AGO2 from neuro2a cells transfected with scrambled siRNA (scr-siRNA) or fmr1 siRNA (fmr1-siRNA). Values are normalized to AGO2 levels in scrambled siRNA (n=3, ±SEM).
3. Validation of absence of FMRP in Fmr1 KO as compared to WT by immunoblotting.
4. Immunoblot for AGO2 and tubulin from neuro2a cells transfected with scramble (scr-siRNA) or AGO2 siRNA (AGO2-siRNA). Distribution of MOV10 separated on a linear sucrose gradient from neuro 2a cells transfected with scramble siRNA (scr-siRNA) or AGO2 siRNA (AGO2-siRNA) based on immunoblots shown below.
5. Quantification of MOV10 in polysomes from neuro 2a cells transfected with scramble siRNA or AGO2 siRNA (n=3, unpaired Student’s t-test, ±SEM) based on immunoblots shown.


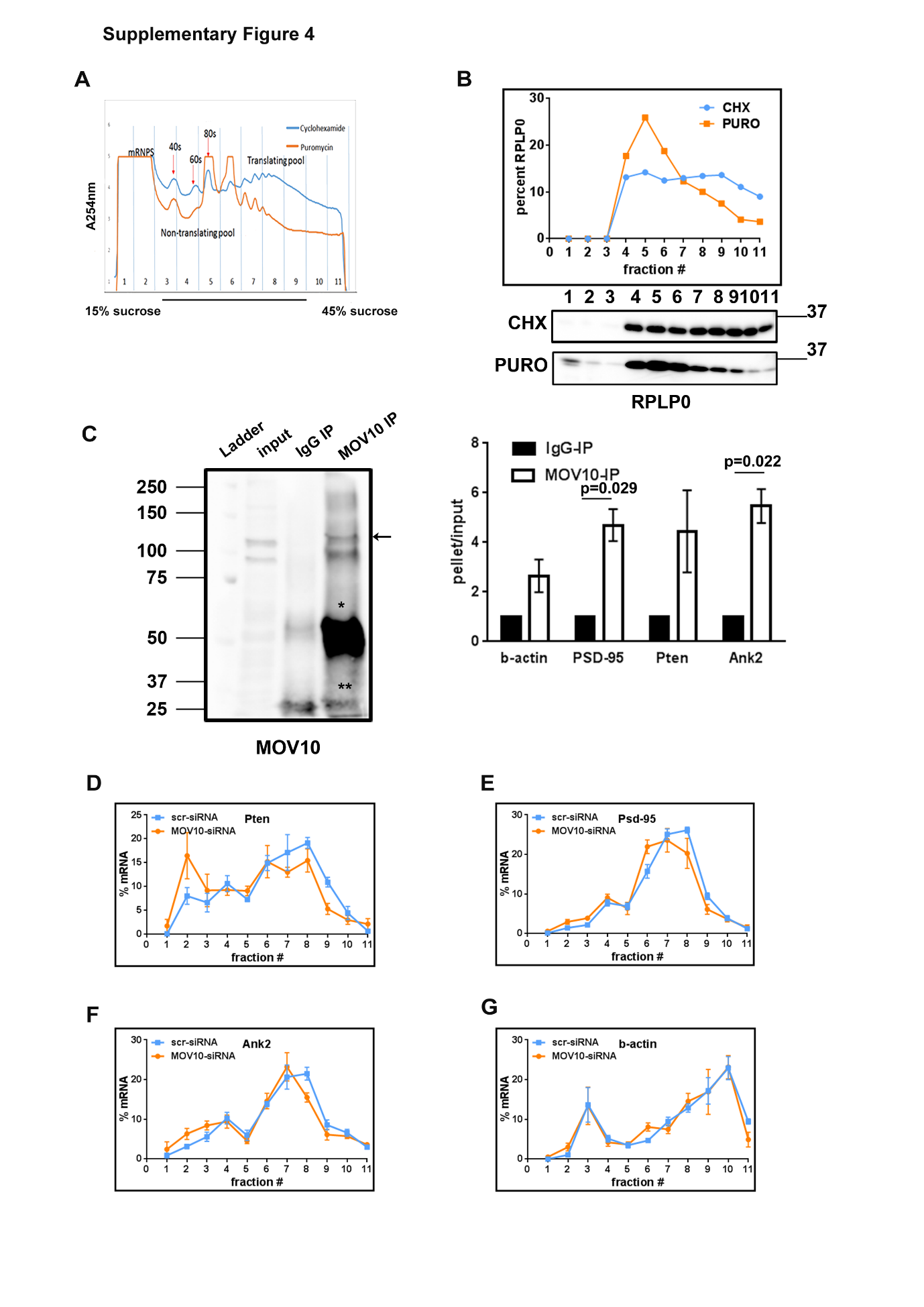


**Supplementary Figure 4:** Puromycin sensitive fractions in primary neurons and validation of MOV10 targets by RNA-IP and (related to Figure 3)

1. ­­A254 trace from primary rat neuronal cultures (DIV 14) separated on a linear sucrose gradient (15-45%) of cycloheximide and puromycin treated.
2. Comparison of ribosome distribution on linear sucrose gradient on cycloheximide and puromycin treatment based on RPLP0 immunoblot (below) from primary rat neuronal cultures (DIV 14).
3. Immunoblot for MOV10 after immunoprecipitation with MOV10 and mouse IgG from primary neurons. Arrow indicates band of interest for MOV10 at ~110kDa, asterisk indicates denatured heavy chain (*) and light chain (**) of IgG. Quantitative analysis of selected mRNAs from MOV10 immunoprecipitation by q-PCR (n=3, paired Student’s t-test, ±SEM). Values are normalized to those in IgG immunoprecipitation.
4. – (G) Average distribution of selected mRNAs on linear sucrose gradient from primary neurons transfected with scrambled siRNA (scr-siRNA) or MOV10 siRNA (MOV10-siRNA) (n=4-6, ±SEM)


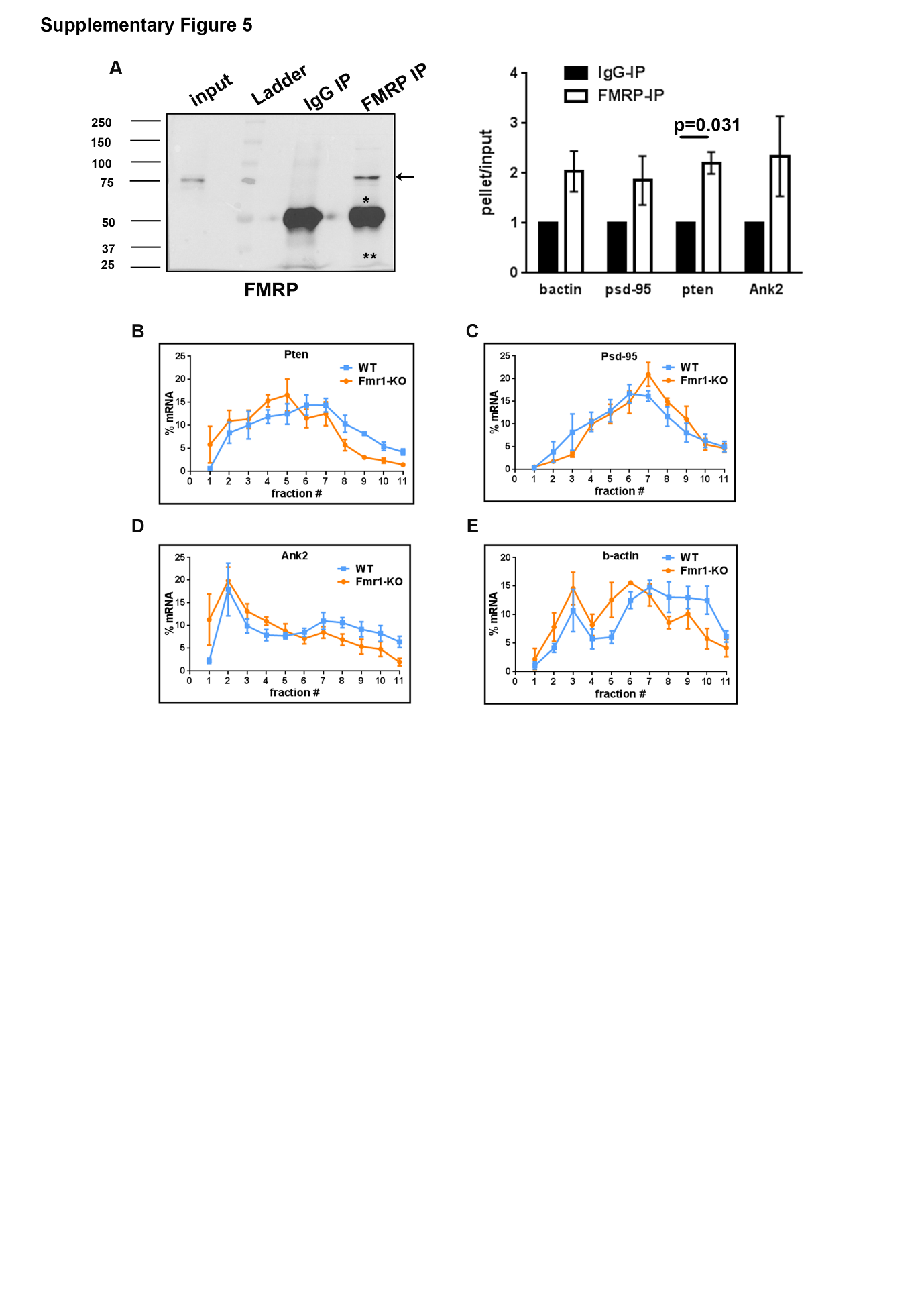


**Supplementary Figure 5**: Validation of FMRP targets by RNA-IP (related to Figure 4)

1. Immunoblot for FMRP after immunoprecipitation with FMRP and rabbit IgG from primary neurons. Arrow indicates band of interest for FMRP at ~75kDa, asterisk indicates denatured heavy chain (*) and light chain (**) of IgG. Quantitative analysis of association of selected mRNAs with mouse IgG or FMRP after IgG or FMRP immunoprecipitation (n=3, paired Student’s t-test, ±SEM). Values are normalized to those in IgG immunoprecipitation.
2. – (E) Average distribution of selected (MOV10 targets) mRNAs on linear sucrose gradient from WT and Fmr1 KO rat synaptoneurosomes (n=3-5, ±SEM)


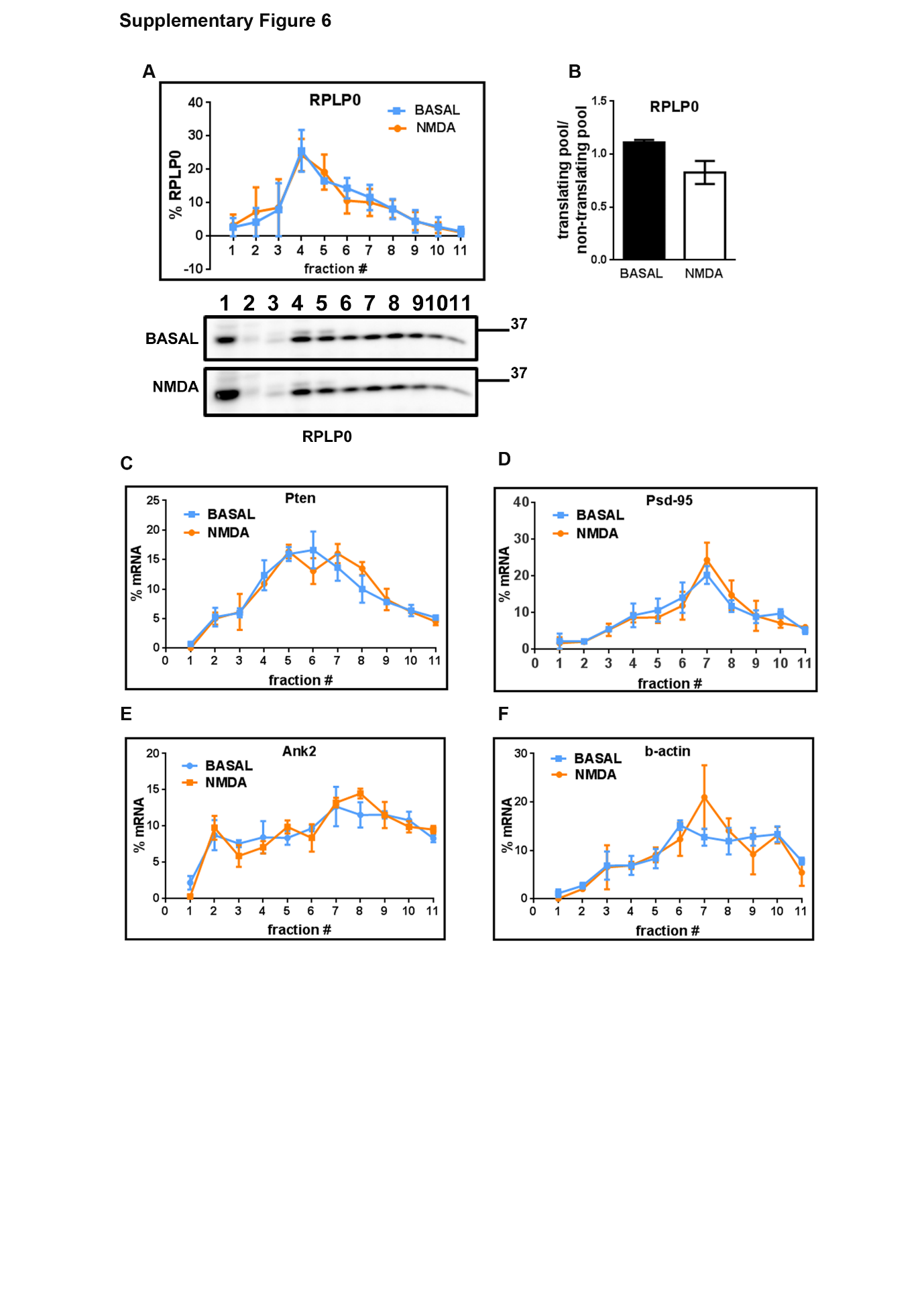


**Supplementary figure 6:** NMDAR stimulation leads to no change in the RPL0 distribution (related to figure 5)

1. Distribution of ribosomal protein RPLP0 on linear sucrose gradient from rat cortical synaptoneurosomes after NMDAR treatment based on immunoblots shown.
2. Quantification of RPLP0 in polysomes from rat synaptoneurosomes treated with NMDA (n=3, ±SEM) based on immunoblots shown.
3. – (F) Average distribution of mRNAs on linear sucrose gradient from synaptoneurosomes at basal state and on NMDAR stimulation (n=3, ±SEM)


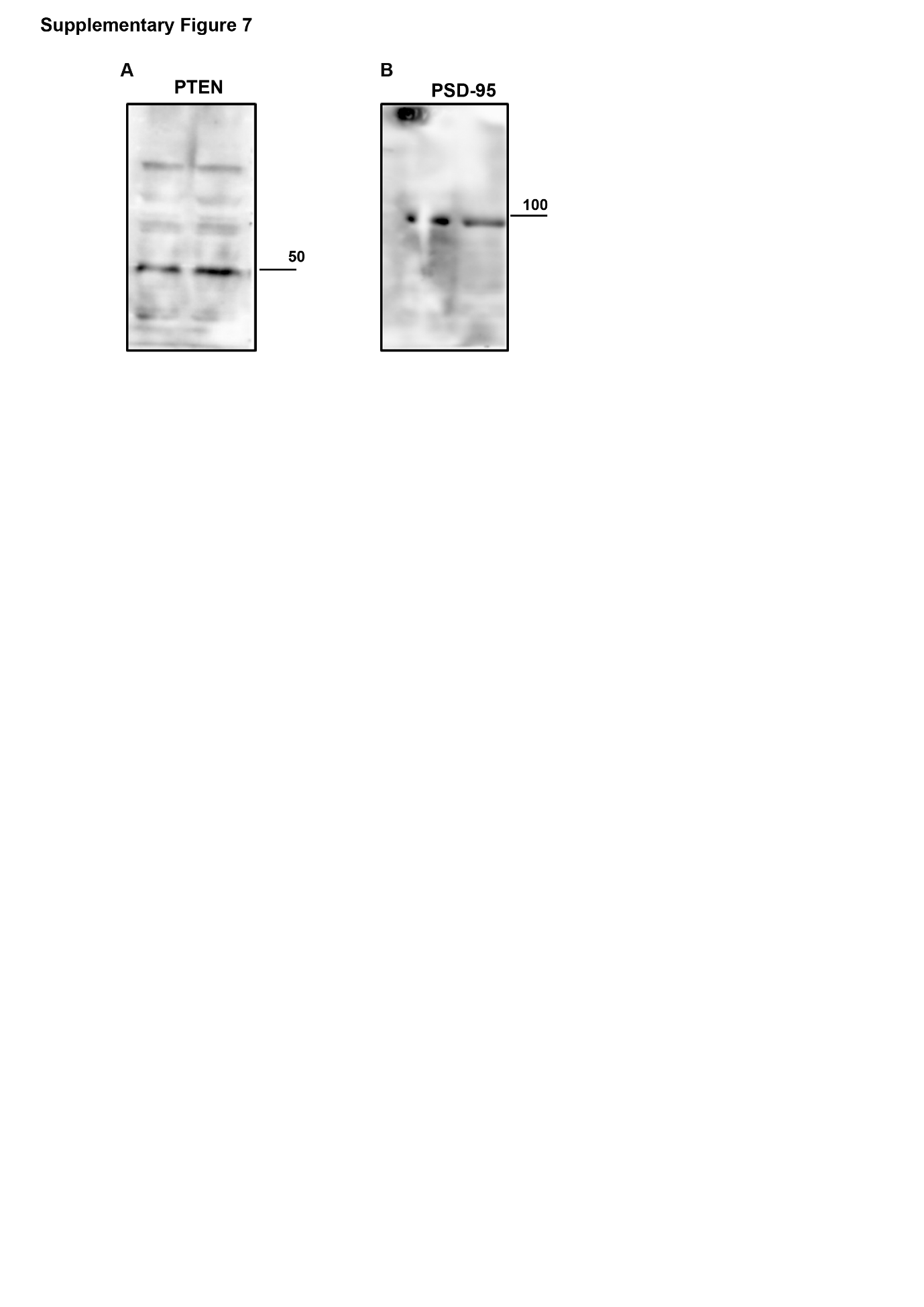


**Supplementary figure 7**: Whole blots for PTEN and PSD-95 to show antibody specificity (related to figure 6)

1. Whole western blot image for anti-PTEN antibody from rat synaptoneurosomes.
2. Whole western blot image for anti-PSD-95 antibody from rat synaptoneurosomes


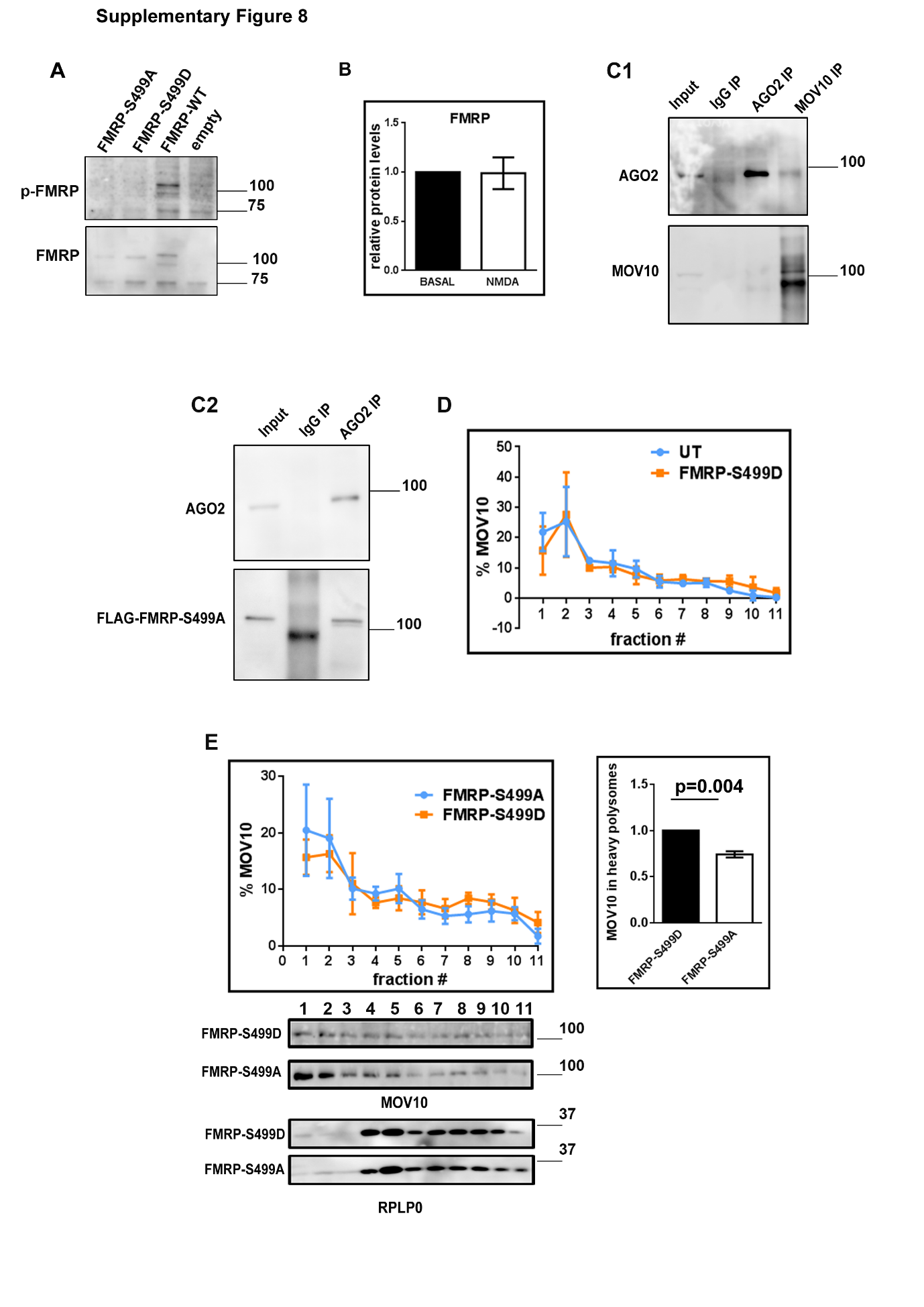


**Supplementary Figure 8**: Phosphorylation of FMRP is the switch for NMDAR mediated translation (Related to Figure 7)

1. Immunoblot for phospho-FMRP from neuro2a cells expressing phosphomimic forms of FMRP, FMRP-S499A or FMRP-S499D or FMRP-WT using anti-phospho FMRP antibody. The same samples were run parallel to probe for endogenous and over-expressed FMRP, with molecular weight indicated (below).
2. Quantification of total FMRP from synaptoneurosomes after NMDAR stimulation. Values are normalized to basal levels (n=6, ±SEM).
3. 1. Immunoblot for AGO2 or MOV10 after IgG IP, AGO2 IP and MOV10 IP.
4. 2. Immunoblot for AGO2 and FLAG-FMRP-S499A after IgG IP or AGO2 IP.
5. Average distribution of MOV10 separated on a linear sucrose gradient from neuro 2a cells untransfected (UT) or transfected with FMRP-S499D (n=3, ±SEM).
6. Average distribution of MOV10 separated on a linear sucrose gradient from neuro 2a cells transfected with FMRP-S499D or FMRP-S499A based on immunoblots shown below followed by quantification of MOV10 in polysomes (n=3, unpaired Student’s t-test, ±SEM). Below: Immunoblot for MOV10 and RPLP0 separated on a linear sucrose gradient from neuro 2a cells transfected with FMRP-S499D or FMRP-S499A.
